# Second-Generation Antipsychotics Induce Metabolic Disruption in Adipose Tissue-Derived Mesenchymal Stem Cells Through an aPKC-Dependent Pathway

**DOI:** 10.3390/cells13242084

**Published:** 2024-12-17

**Authors:** Marco Varalda, Jacopo Venetucci, Herald Nikaj, Chaitanya Reddy Kankara, Giulia Garro, Nazanin Keivan, Valentina Bettio, Paolo Marzullo, Annamaria Antona, Guido Valente, Sergio Gentilli, Daniela Capello

**Affiliations:** 1 Department of Translational Medicine, Centre of Excellence in Aging Sciences, University of Piemonte Orientale, 28100 Novara, Italy; jacopo.venetucci@uniupo.it (J.V.); 20041555@studenti.uniupo.it (C.R.K.); giulia.garro@uniupo.it (G.G.); 20047723@studenti.uniupo.it (N.K.); valentina.bettio@med.uniupo.it (V.B.); paolo.marzullo@med.uniupo.it (P.M.); annamaria.antona@uniupo.it (A.A.); guido.valente@med.uniupo.it (G.V.); sergio.gentilli@med.uniupo.it (S.G.); daniela.capello@med.uniupo.it (D.C.); 2 UPO Biobank, University of Piemonte Orientale, 28100 Novara, Italy; 3 General Surgery Division, University of Piemonte Orientale, AOU Maggiore della Carità, 28100 Novara, Italy; 20027483@studenti.uniupo.it; 4 Pathology Unity, Ospedale “Sant’Andrea”, 13100 Vercelli, Italy; 5 Department of Health Sciences, University of Piemonte Orientale, 28100 Novara, Italy

**Keywords:** second-generation antipsychotics, insulin resistance, adipose tissue, adipose-derived mesenchymal stem cells, insulin signaling, endocytosis, lysosomes, PKCζ

## Abstract

Metabolic syndrome (MetS) is a cluster of metabolic abnormalities, including visceral obesity, dyslipidemia, and insulin resistance. In this regard, visceral white adipose tissue (vWAT) plays a critical role, influencing energy metabolism, immunomodulation, and oxidative stress. Adipose-derived stem cells (ADSCs) are key players in these processes within vWAT. While second-generation antipsychotics (SGAs) have significantly improved treatments for mental health disorders, their chronic use is associated with an increased risk of MetS. In this study, we explored the impact of SGAs on ADSCs to better understand their role in MetS and identify potential therapeutic targets. Our findings reveal that olanzapine disrupts lipid droplet formation during adipogenic differentiation, impairing insulin receptor endocytosis, turnover, and signaling. SGAs also alter the endolysosomal compartment, leading to acidic vesicle accumulation and increased lysosomal biogenesis through TFEB activation. PKCζ is crucial for the SGA-induced nuclear translocation of TFEB and acidic vesicle formation. Notably, inhibiting PKCζ restored insulin receptor tyrosine phosphorylation, normalized receptor turnover, and improved downstream signaling following olanzapine treatment. This activation of PKCζ by olanzapine is driven by increased phosphatidic acid synthesis via phospholipase D (PLD), following G protein-coupled receptor (GPCR) signaling activation. Overall, olanzapine and clozapine disrupt endolysosomal homeostasis and insulin signaling in a PKCζ-dependent manner. These findings highlight SGAs as valuable tools for uncovering cellular dysfunction in vWAT during MetS and may guide the development of new therapeutic strategies to mitigate the metabolic side effects of these drugs.

## 1. Introduction

Metabolic syndrome (MetS) is a term used to define a cluster of metabolic abnormalities that include visceral obesity, hypertension, dyslipidemia, and insulin resistance (IR) [1,2]. The worldwide spread of obesity has become a critical public health problem due to its strong association with type 2 diabetes (T2D) and cardiovascular diseases (CVDs) [3,4]. Visceral white adipose tissue (vWAT) is a highly dynamic endocrine and immune organ that is heavily involved in the biological processes that occur during MetS. This is because of this tissue’s systemic influence on energy metabolism, immunomodulation, and oxidative stress [5,6,7]. During MetS, vWAT undergoes significant modifications in terms of abundance, cellular composition, and metabolism, leading to hypertrophy and hyperplasia of adipocytes, modification in the secretome of adipokines and proinflammatory cytokines, and IR, with or without glucose tolerance [6,8,9]. WAT primarily comprises two cellular populations: mature adipocytes and the stromal vascular fraction (SVF). The latter includes a diverse range of cells such as endothelial cells, fibroblasts, macrophages, preadipocytes, and mesenchymal stem cells (MSCs), also known as adipose-derived stem cells (ADSCs) [10].

In recent years, ADSCs have garnered significant attention due to their therapeutic potential in managing diabetes, inflammatory disorders, and age-related diseases [11,12,13,14]. The ADSC secretome has been identified as a key player in exerting these therapeutic benefits, due to its role in regulating essential biological processes such as inflammation, oxidative stress, and angiogenesis [15,16,17]. In clinical settings, autologous ADSC therapy has shown promising results, including improved glucose tolerance and enhanced insulin sensitivity, in diabetic patients [12,18,19]. Moreover, recent research has increasingly focused on understanding how changes in ADSC function may contribute to the pathogenesis of systemic diseases.

In the context of T2D and MetS, studies have documented significant disruptions in ADSC differentiation, cytokine production, and angiogenic capacity, positioning ADSCs as a vital model for unraveling the molecular mechanisms underlying MetS [20,21,22,23,24].

Antipsychotics are a class of drugs representing the first line of treatment for many psychiatric disorders such as schizophrenia and bipolar disorder [25,26]. Second-generation antipsychotics (SGAs), including olanzapine, clozapine, risperidone paliperidone, and aripiprazole, are psychotropic drugs that were developed in the 1990s. They represent a distinct improvement compared to first-generation antipsychotics due to their weaker extrapyramidal side effects [27,28,29]. Despite these therapeutic advantages, SGAs are associated with substantial metabolic side effects, including MetS, which can develop within the first six weeks of treatment and impacts approximately 10–20% of patients [30]. This condition markedly increases the incidence of obesity, cardiovascular disease (CVD), and T2D in these patients [26]. While lifestyle factors such as enhanced food intake have been implicated, recent findings suggest that SGAs have a direct role in adipocyte function dysregulation [31,32]. They can disrupt lipid storage and adipokine secretion—key processes for metabolic stability—leading to fat accumulation, impaired insulin signaling, an overall pro-inflammatory state, and ultimately fostering MetS onset. Therefore, investigating the mechanisms underlying SGA-induced metabolic disturbance is essential for advancing our understanding of the pathophysiology of MetS.

The primary objective of this study was to investigate the impact of SGAs, specifically olanzapine and clozapine, on visceral ADSCs to understand the molecular pathways affected by these drugs. Our work demonstrates the potential of SGAs as a tool for identifying cellular dysfunctions in vWAT linked to MetS. These results offer a unique contribution in bridging the gap between psychopharmacology and metabolic research, providing valuable insights into the mechanisms of SGA-induced MetS and identifying new therapeutic targets. These insights can inform the development of strategies to mitigate the metabolic side effects associated with SGAs, enriching our understanding of adipose tissue dysregulation in MetS.

## 2. Materials and Methods

### 2.1. Reagents

The SGAs—olanzapine and clozapine—were purchased from Cayman Chemical Company (Cayman Chemicals, Ann Arbor, MI, USA), dissolved in DMSO (Sigma-Aldrich, Saint Louis, MO, USA), and stored at –20°. The differentiation factors—insulin, dexamethasone, and rosiglitazone—were purchased from the same company and received the same treatment. IBMX was also bought from Cayman Chemicals, dissolved in ethanol, and stored at –20 °C. The PKCζ-pseudo substrate myristoyl trifluoroacetate salt was purchased from Sigma-Aldrich (Sigma-Aldrich, Saint Louis, MO, USA) and dissolved in H_2_O. Fipi (PLD1 inhibitor), EPAC inhibitor, SQ22 (adenylate cyclase inhibitor), suramin, YM254890 (Gaq/11 inhibitor), Go5860 (PKC inhibitor), and R59022 were also purchased from Cayman Chemical Company. Lysotracker Deep Red was bought from Invitrogen (Invitrogen, Waltham, MA, USA), while Hoechst 33342 was bought from Sigma (Sigma-Aldrich, Saint Louis, MO, USA) and dissolved in H_2_O at a concentration of 5 mg/mL.

For Western blot and immunofluorescence analyses, the primary antibodies were anti-Tubulin, anti-P-AKT S473, anti-P-AKT T308, anti-AKT, LAMP1 (Santa Cruz Biotechnology, Dallas, TX, USA) LC3B, (Sigma-Aldrich, Saint Louis, MO, USA), anti-P-INSRβ Y1146, anti-INSRβ, anti-P-ERK1/2, anti-ERK1/2, anti-RAB7, anti-P-(Ser)-PKC substrate (Cell Signaling Technology, Danvers, MA, USA), anti-p62, anti-PKCζ (Thermo Fisher Scientific, Waltham, MA, USA), anti-p-PKCζ T560 (Abcam, Cambridge, UK), and anti-CD63 (BD Biosciences, Franklin Lakes, NJ, USA). The secondary antibodies were goat anti-mouse IgG or donkey anti-rabbit IgG horseradish peroxidase (HRP); these were purchased from Perkin Elmer (PerkinElmer, Waltham, MA, USA). For immunofluorescence microscopy experiments, Phalloidin 546, Phalloidin 633, and secondary antibodies, Alexafluor 488 and 564, were bought from Thermo Fisher Scientific (Thermo Fisher Scientific, Waltham, MA, USA).

### 2.2. Adipose-Derived Mesenchymal Stem Cell Isolation and Expansion

Primary vWAT biopsy samples were collected in collaboration with the General Surgery Department of Ospedale Maggiore della Carità of Novara from informed patients participating in the ADAGE Study (approved by the Novara Ethical Committee, CE 10-21, 14 January 2021). The samples were washed with PBS 1X and mechanically fragmented into small pieces, approximately 1 mm in size, using sterile scalpels. The fragmented tissue was then digested with collagenase Type I (1 mg/mL, Sigma-Aldrich, Saint Louis, MO, USA) for 60 min at 37 °C with continuous shaking. Following enzymatic digestion, the stromal vascular fraction (SVF) was collected and plated in DMEM supplemented with 10% FBS (Euroclone, Milan, Italy) and 1% antibiotics and antimycotics. The cells were cultured for at least three weeks to obtain a homogeneous population of adipose tissue-derived MSCs (ADSCs). The cells were then characterized using flow cytometry in order to evaluate the expression of stemness markers, CD105 and CD73. These cells were stained with primary antibodies, anti-CD105 and anti-CD 73, and analyzed at FACS (Attune NxT, Flow Cytometer, Thermo Fisher Scientific, Waltham, MA, USA) (Supplementary Figure S1).

### 2.3. Cell Culture

Primary ADSCs isolated from human vWAT samples and mouse preadipocyte 3T3-L1 cell lines (purchased from ATCC) were used for the experiments. These cells were cultured in Dulbecco’s Modified Eagle Medium (DMEM, Gibco, Waltham, MA, USA) with 10% Fetal Bovine Serum (FBS, Euroclone, Milan, Italy) and 1% antibiotics–antimycotics (Penicillin, Streptomicyn, Amphotericyn, Sigma, SaintLouis, MO, USA) at a controlled temperature and atmosphere (37 °C, 5% CO_2_).

### 2.4. Differentiation of ADSCs in White Adipocytes

ADSCs were plated at a density of 70,000 cells/well in a 12-well plate using DMEM with 10% FBS. When the cells reached about 90% confluence, the culture medium was removed and substituted with WAT-differentiating medium containing DMEM 10% FBS, insulin (1 μM), dexamethasone (DEXA, 1 μM), IBMX (0.5 mM), and rosiglitazone (1 μmol/mL). After 3 days of exposure to the differentiating medium, the cells were cultured in a maintenance medium containing only insulin (1 μM) and rosiglitazone (1 μM) for 14 days. The medium was changed every 3 days. The differentiation results were evaluated by staining the cells with HCS LipidTOX™ for neutral lipids (Thermo Fisher Scientific, Waltham, MA, USA) after 21 days. The cells were fixed using 4% paraformaldehyde (PFA, Sigma-Aldrich, SaintLouis, MO, USA) for 15 min at room temperature; then, each well was stained using HCS LipidTox and Hoechst 33342 and incubated at room temperature for 30 min. After this incubation, the cells were washed with water. Images were acquired using fluorescence microscopy (FLoid Cell Imaging Station, Life Technology, Carlsbad, CA, USA) or light microscopy (DMi1, Leica, Wetzlar, Germany) and analyzed using ImageJ software (v 1.52a).

### 2.5. Cell Viability

For each cell line, 1000 cells/well were plated in a 96-well plate. The cells were treated with different concentrations of drugs and incubated for either 3 or 7 days. For each drug concentration, the same concentration of vehicle (DMSO) was used as a control. During plating, the cells were stained with CellTox Green reagent (Promega, Madison, WI, USA) according to the manufacturer’s instructions. Cell viability was assessed using a Victor multilabel plate reader X4 (Perkin Elmer, Waltham, MA, USA) by measuring the fluorescence signal at 485–510 nm, as outlined in the manufacturer’s instructions.

### 2.6. Acidic Vesicle Quantification

ADSCs and 3T3-L1 cells were plated at a density of 10,000 cells per well in a 48-well plate and treated with varying concentrations of olanzapine and clozapine (0.5, 1, 2.5, 5, 10 µM) for 24 h, 72 h, and 7 days. After treatment, the medium was removed, and the cells were stained with 50 nM Lysotracker Deep Red (Invitrogen, Waltham, MA, USA) and 5 μg/mL Hoechst 33342 (Sigma). Staining was conducted in the dark at 37 °C for 30 min. Fluorescence signals were captured using a FLoid Cell Imaging Station (Life Technology, Carlsbad, CA, USA). The ratio of Lysotracker red signal to blue nuclear signal was quantified using ImageJ software. For acidic vesicle rescue experiments, ADSCs were plated at a concentration of 10,000 cells per well in 48-well plates. The cells were treated with olanzapine or clozapine (5 µM), either alone or in combination with one of the following treatments: 3-methyladenine (3-MA; 1 mM), CHX (10 µg/mL), Go6850 (1 µM), PKCζ-pseudo substrate myristoyl trifluoroacetate salt (10 µM), Fipi (750 nM), EPAC inhibitor (10 µM), SQ22 (10 µM), suramin (10 µM), or YM254890 (10 µM).

### 2.7. Vacuolization Assay

ADSCs were plated at a density of 10,000 cells/well in a 48-well plate and then treated with olanzapine, clozapine, fluoxetine, or ebastine at a concentration of 5 μM. After 4 h, pictures were acquired using a phase contrast microscope (DMi1, Leica, Wetzlar, Germany).

### 2.8. Phospholipidosis Assay

ADSCs were plated at a density of 10,000 cells/well in a 48-well plate and treated with 5 μM of either olanzapine, clozapine, or fluoxetine. The cells were stained with 1X LipidTox Green (Thermo Fisher Scientific, Waltham, MA, USA) for 16 h to visualize lipid content. Following lipid staining, the nuclei were stained with 5 μg/mL Hoechst 33342, and the plate was incubated in the dark at 37 °C for 30 min. The cells were then washed with PBS and fixed with 4% paraformaldehyde for 15 min in the dark. Fluorescence signals were acquired using a FLoid Cell Imaging Station (Life Technology), and the images were analyzed with ImageJ software (v 1.52a).

### 2.9. SDS-PAGE/Western Blot

After treatments, whole-cell lysates were prepared using RIPA lysis buffer supplemented with protease inhibitors (AEBSF, aprotinin, bestatin, E-64, EDTA, leupeptin, Sigma-Aldrich, Saint Louis, MO, USA) and orthovanadate (Sigma-Aldrich, Saint Louis, MO, USA). The proteins in the lysates were collected and quantified using the Pierce BCA protein assay kit (Thermo Fisher Scientific, Waltham, MA, USA). Subsequently, the proteins were denatured by heating at 95 °C for 5 min in the presence of 2% Sodium Dodecyl Sulfate (SDS, Sigma-Aldrich, Saint Louis, MO, USA), 150 mM dithiothreitol (DTT, Sigma-Aldrich, Saint Louis, MO, USA), and 0.01% bromophenol blue (Sigma-Aldrich, Saint Louis, MO, USA). The samples were then analyzed using SDS-PAGE (SDS-Polyacrylamide Gel Electrophoresis) and Western blotting (WB).

### 2.10. Immunoprecipitation Assay

ADSCs were seeded at a density of 100,000 cells/well in a 6-well plate and treated with olanzapine at 5 μM for 16 h. Following treatment, the cells were stimulated with insulin at 50 ng/mL for 15 min. For the immunoprecipitation of the insulin receptor beta subunit (INSRβ), cell lysates were prepared using RIPA lysis buffer with protease inhibitors, as described previously. The lysates were then incubated for 1 h with magnetic beads (Dynabeads Protein G, Thermo Fisher Scientific, Waltham, MA, USA) that had been previously bound to anti-INSRβ antibody, following the manufacturer’s instructions. After incubation, the beads were washed three times with RIPA buffer to remove non-specifically bound proteins. The immunoprecipitated proteins bound to the beads were then eluted by boiling the beads at 95 °C for 5 min in the presence of 2% SDS, 150 mM DTT, and 0.01% bromophenol blue. The eluted proteins were analyzed using SDS-PAGE and, subsequently, using WB.

### 2.11. Endocytosis Assay

ADSCs were seeded at a density of 100,000 cells/well in a 6-well plate. The protocol was carried out as described in Barrow-McGee et al., 2015 [33]. Cell surface proteins were labeled by incubating the cells with 0.2 mg/mL sulpho-NHS-SS-biotin (Sigma-Aldrich, Saint Louis, MO, USA) in PBS for 45 min on ice. Labeled cells were then washed with cold PBS and incubated at 37 °C in a culture medium containing insulin at 50 ng/mL to allow for INSR trafficking. At the indicated times, the medium was removed and the dishes were transferred to ice and washed with cold PBS. Biotin was removed from the proteins that remained at the cell surface via reduction for 15 min with 180 mM of the membrane-impermeant reducing agent MesNa (sodium 2 mercaptoethane sulphonate, Sigma-Aldrich, Saint Louis, MO, USA) in 50 mM Tris and 100 mM NaCl at pH 8.6. MesNa was quenched by adding 180 mM iodoacetamide (IAA, Sigma-Aldrich, Saint Louis, MO, USA) for 10 min. The cells were lysed using RIPA buffer as previously described. The proteins were quantified using BCA; equal protein amounts received streptavidin–agarose beads (Sigma-Aldrich, Saint Louis, MO, USA) and were agitated at 4 °C for 2 h. Then, the beads were collected via centrifugation (7000× *g*) and washed in lysis buffer, and the proteins were extracted by heating at 95 °C with sample buffer. In each internalization assay, two controls were carried out. To measure INSRβ at the surface, biotinylated cells at 4 °C were lysed without biotin reduction (Ts). To verify the efficiency of the surface biotin removal, the biotin reduction and MesNa-quenching steps were carried out on cells that had remained on ice (T0). Then, lysis was carried out. All lysates were analyzed via Western blot.

The percentages of internalized INSR were calculated using the following formula: internalized receptor = (receptor level after incubation at 37 °C) − (receptor level at time 0)/(total surface receptor) × 100. All samples were normalized on the input INSR signal.

### 2.12. Immunofluorescence

ADSCs were seeded at a density of 10,000 cells/well on glass coverslips and treated with 5 μM olanzapine or clozapine for 16 h. For INSRβ endocytosis experiments, the cells were stimulated with 50 ng/mL insulin for 15 min. For transferrin endocytosis experiments, the cells were incubated with 25 μg/mL Transferrin Texas Red for 5 min. After treatment, the cells were washed with PBS and fixed with 4% PFA for 10 min at room temperature, followed by an additional PBS wash. The cells were then permeabilized by incubating them with cold HEPES-Triton X-100 buffer for 5 min at 4 °C. After permeabilization, the cells were incubated with primary antibodies for 30 min, followed by incubation with Alexa Fluor 488- or 546-conjugated secondary antibodies (Invitrogen, Waltham, MA, USA) and phalloidin, labeled with either Alexa Fluor 546 or 633 (Invitrogen Waltham, MA, USA) for 30 min. After incubation, coverslips were mounted on glass slides using a mounting medium. Images were acquired using a confocal microscope (Leica TCS SP8, Wetzlar, Germany) and analyzed using ImageJ (v 1.52a) software in order to quantify and analyze the fluorescence signals.

### 2.13. SiRNA Transfection

ADSCs were transfected with 30 pmol of either Silencer Select Negative Control siRNA (Thermo Fisher Scientific, Waltham, MA, USA) or Silencer Select Pre-Designed siRNA (Thermo Fisher Scientific, Waltham, MA, USA) targeting PKCζ. Transfection was carried out using Lipofectamine 3000 (Thermo Fisher Scientific, Waltham, MA, USA) according to the manufacturer’s instructions. After transfection, the cells were incubated with the siRNA for 24 h and used for experiments 72 h post-transfection.

### 2.14. Plasmid Transfection

3T3-L1 cells were transfected with 5 μg of the Pii-PA (PA indicator with superior sensitivity) DOCK2 plasmid (DOCK2-Pii) [34]. Transfection was carried out using Lipofectamine 3000 (Thermo Fisher Scientific, Waltham, MA, USA), following the manufacturer’s instructions. The cells were incubated with the plasmid for 24 h to ensure successful transfection and plasmid expression and were used for experiments 48 h post-transfection.

### 2.15. RNA Extraction and Real-Time PCR

After treatment, RNA was extracted using the phenol/chloroform method (RNAzol, Sigma-Aldrich, Saint Louis, MO, USA) followed by isopropanol precipitation, according to the manufacturer’s instructions. The precipitated RNA was then washed with 75% ice-cold ethanol and resuspended in water. The RNA concentration was measured using NanoDrop 2000. cDNA was synthesized via reverse transcription using recombinant Moloney murine leukemia virus reverse transcriptase (MultiScribe Reverse Transcriptase, Bio-Rad, Hercules, CA, USA) and the iScript cDNA Synthesis Kit (Bio-Rad, Hercules, CA, USA). Gene expression was analyzed using real-time PCR using the SsoAdvanced Universal SYBR Green Supermix Kit (Bio-Rad, Hercules, CA, USA). The target genes are listed in Table 1. Relative quantification was calculated using the ΔΔCt method [35].

### 2.16. Statistical Analysis

Statistical analyses and graph generation were performed using GraphPad Prism 8.0 software (GraphPad Software Inc., San Diego, CA, USA). For viability assays, IC50 values were calculated using a variable slope model based on assay data, and a semi-logarithmic dose–response curve was generated. When comparing between two groups, statistical significance was assessed using Student’s *t*-test, with a significance threshold set at *p* < 0.05. The Pearson coefficient was used to assess the spatial relationship between the proteins under investigation; this was calculated using the JacoP plugin of ImageJ. The coefficient ranges from +1 to −1, where a value of +1 indicates a perfect positive correlation (high colocalization), −1 indicates a perfect negative correlation (mutually exclusive localization), and values close to 0 suggest no significant correlation between the proteins.

## 3. Results

### 3.1. ADSC Viability Is Not Affected by Therapeutic Concentrations of SGAs

**Figure 1:**
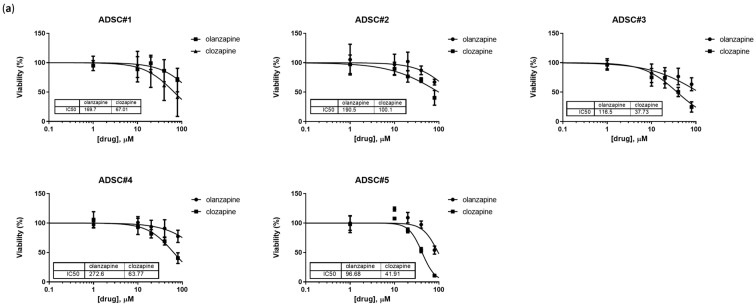

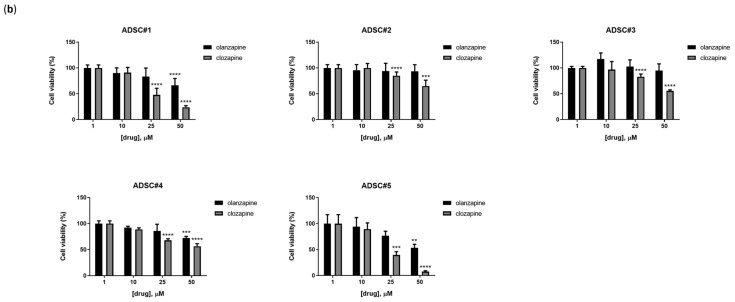
Assessment of olanzapine and clozapine cytotoxic activity in ADSCs. Viabilities of ADSCs treated with scalar doses of drugs for 72 h; IC50, i.e., we calculated the drug concentration reduced by 50% in terms of viability compared to the control (**a**). Bar graphs showing cell viability after 7 days of treatment with scalar doses of drugs; viability data are presented as the percentage of viable cells relative to the negative control treated with DMSO. Data are presented as mean ± SEM from three independent experiments (**b**). **, Student’s *t*-test *p* < 0.01; ***, Student’s *t*-test *p* < 0.001; ****, Student’s *t*-test *p* < 0.001.

Evidence demonstrates that, in MetS and T2D, ADSC fitness declines, along with widespread disruptions in vWAT homeostasis [36]. Considering that SGAs have been reported to affect WAT function in both animal and human models [32,37], we initially questioned whether the metabolic dysregulation caused by these compounds involved direct cytotoxic effects on ADSCs. To address this hypothesis, cells were exposed for 72 h to olanzapine and clozapine at concentrations ranging from 1 μM to 160 μM. While high, these levels are consistent with those used in prior studies investigating these compounds. Our results revealed that olanzapine reduced cell viability by 50% only at concentrations exceeding 95.0 μM in all tested cell lines, while clozapine exhibited slightly higher cytotoxicity, with an IC50 of 37.8 μM in the most sensitive cell line (Figure 1a, Supplementary Figure S2). These findings indicate that cytotoxicity occurs only at doses much higher than those typically encountered in patients. Next, we evaluated whether chronic exposure at lower doses might impact ADSC viability. ADSCs were treated with drug concentrations ranging from 1 to 50 μM over 7 days. Even under these prolonged conditions, observed IC50 values remained significantly higher than the plasma concentrations commonly observed in patients receiving chronic treatment (0.5 to 2 µM) [38,39]. Olanzapine exhibited negligible effects on cell viability under all tested conditions, whereas clozapine showed significant toxicity at concentrations above 25 μM (Figure 1b). Based on these observations, we selected a working concentration of 5 µM for subsequent experiments. This choice allows us to exclude non-specific cytotoxic effects while reflecting concentrations potentially achievable within the AT microenvironment. This approach ensures that we can observe metabolic effects relevant to long-term SGA treatment, facilitating a more clinically relevant comparison and minimizing non-specific off-target effects from excessive, non-physiological drug concentrations.

### 3.2. Olanzapine Impairs the Adipogenic Differentiation of ADSCs

**Figure 2:**
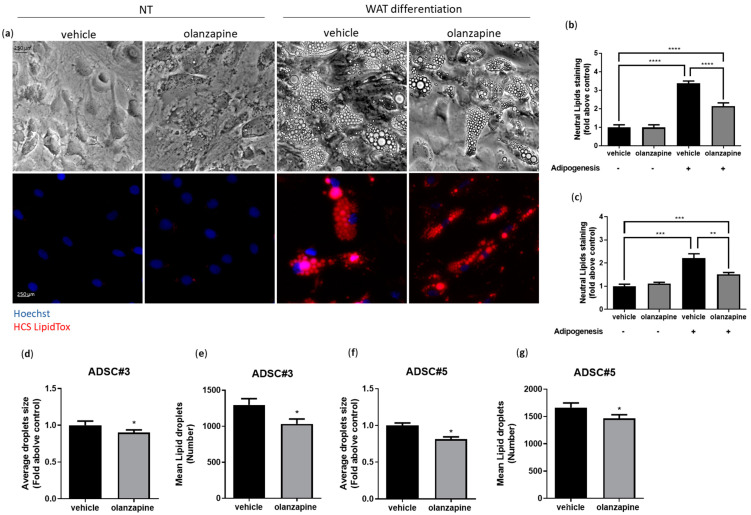
Olanzapine affects ADSC adipogenic differentiation. Representative images of lipid droplets in ADSC#3 treated with 5 μM olanzapine alone or in combination with WAT-differentiating medium using HCS LipidTox for neutral lipids; nuclei were stained using Hoechst 33342 (**a**). Bar graphs showing quantification of lipid droplet staining/blue nuclei staining ratio as fold change relative to control in ADSC#3 (**b**) and ADSC#5 (**c**); data are expressed as the mean ± SD of a representative experiment out of three independent experiments performed in triplicate. Graphs showing quantification of mean dimension and number of lipid droplets in ADSCs treated with olanzapine and in controls (**d**–**g**); data are expressed as the mean ± SD of a representative experiment out of three independent experiments. *, Student’s *t*-test *p* < 0.05. **, Student’s *t*-test *p* < 0.01 ***, Student’s *t*-test *p* < 0.001. ****, Student’s *t*-test *p* < 0.0001.

Adipogenesis inhibition is a critical factor in disrupting vWAT homeostasis [40,41]. This prompted us to examine the effects of olanzapine on ADSC differentiation. ADSCs were exposed to 5 µM olanzapine or vehicle for 24 h before incubation in the differentiation medium. Exposure was continued throughout the differentiation process. After three weeks, staining for neutral lipid droplets revealed a phenotype consistent with adipogenic differentiation, both in controls and treated cells, but with a significant reduction of approximately 30% in lipid accumulation in olanzapine-treated adipocytes (Figure 2a–c), with both the quantity and average size of lipid droplets being notably decreased (Figure 2d–g). We investigated the expression of genes related to adipogenesis, such as PPARG, FABP4, HSL, and LPL, and we observed a significantly reduced expression in differentiated cells cotreated with olanzapine (Supplementary Figure S3).

### 3.3. Olanzapine Impairs INSR Signaling and Turnover

**Figure 3:**
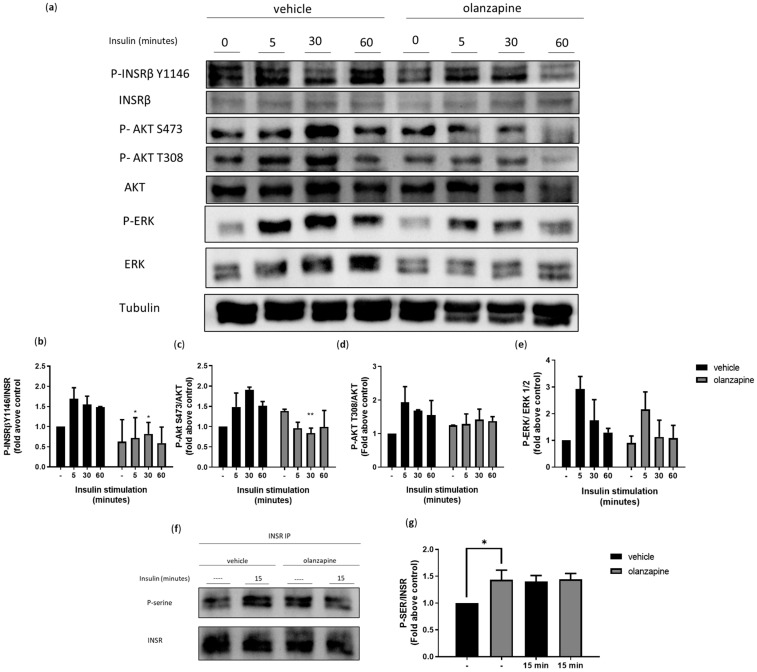
Olanzapine downregulates insulin signaling. Representative Western blot of ADSC#3 cells after 16-h pretreatment with 5 μM olanzapine and stimulation with insulin (50 ng/mL) for 5, 30, and 60 min; lysates were analyzed for P-INSRβ Y1146, INSRβ, P-AKT T308, P-AKT S473, total AKT, P-ERK T202/Y204, and ERK (**a**). Bar graphs showing quantification of P-INSRβ Y1142 (**b**), P-AKT T308 (**c**), P-AKT S473 (**d**), and P-ERK T202/Y204 (**e**) normalized on their respective total proteins and expressed as fold change relative to control. Western blot analysis of immunoprecipitated INSRβ P-Ser in cells stimulated with insulin 50 ng/mL for 15 min (**f**). Bar graph showing quantification of P-Ser signals normalized on total INSR; densitometry expressed as fold change relative to control (**g**). Graphs are expressed as the mean ± SD of three independent experiments. *, Student’s *t*-test *p* < 0.05; **, Student’s *t*-test *p* < 0.01.

Insulin modulates various aspects of WAT function and stimulates adipocyte differentiation [42]. The impaired ADSCs adipogenic differentiation observed with olanzapine treatment prompted us to investigate the response to insulin stimulation. A Western blot analysis of insulin signaling in ADSCs exposed to olanzapine or vehicle for only 16 h showed a reduction in insulin receptor beta (INSRβ) tyrosine phosphorylation (P-INSRβ Y1146), as well as a decrease in P-AKT T308, P-AKT S473, and P-ERK T202/Y204, indicating an overall diminished response to insulin in olanzapine-treated cells (Figure 3a–e). Moreover, increased levels of phosphorylated INSRβ serine residues could be seen in the olanzapine-treated cells. This increase was noticeable not only after insulin stimulation, as expected, but also under basal conditions, suggesting that olanzapine triggers the negative regulation of insulin signaling (Figure 3f,g).

**Figure 4:**
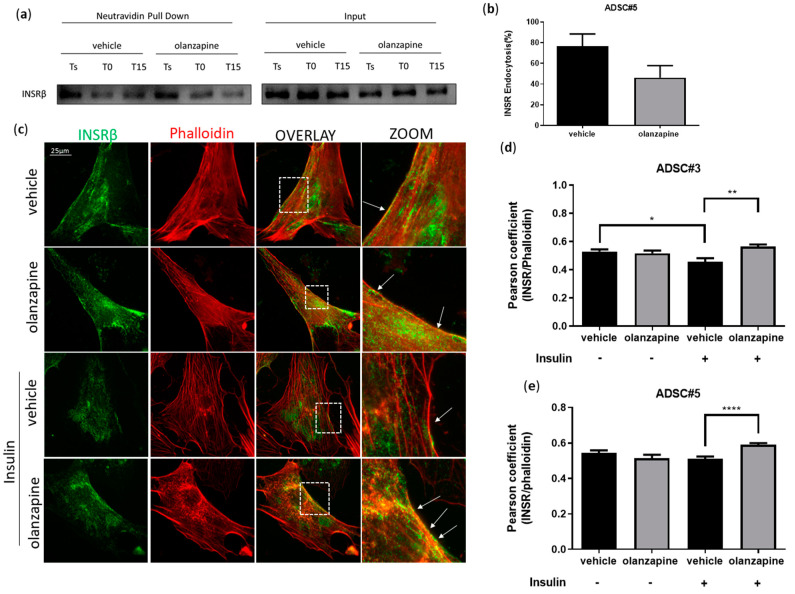

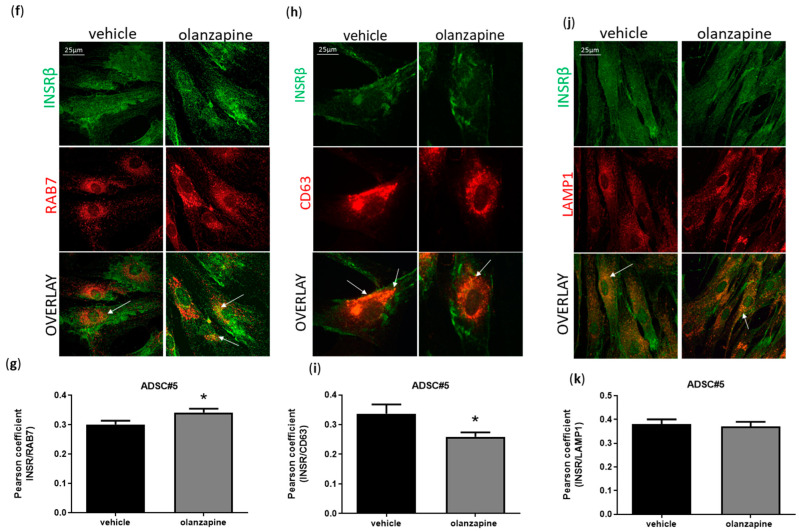
Olanzapine impairs INSR endocytosis. Western blot analysis showing internalization of biotinylated INSRβ in ADSC#5 treated for 16 h with olanzapine and stimulated with insulin for 15 min: Ts represents total biotinylated proteins on the surface, T0 the surface proteins after quenching of the membrane in unstimulated cells, and T15 the surface proteins after endocytosis (**a**). Bar graph representative of 3 independent experiments showing quantification of internalized receptor; densitometry is expressed as T15 /Ts ratio normalized on total INSRβ, as fold change relative to control (**b**). Representative images of INSRβ localization on ADSC#5 plasma membrane after 16 h olanzapine treatment and 15 min insulin stimulation; INSRβ was stained using anti-INSRβ primary antibody and secondary Alexa Fluor 488 (green), while actin was stained using phalloidin 546 (**c**). Bar graphs showing colocalization of INSRβ and actin on ADSC#3 (**d**) and ADSC#5 (**e**) plasma membrane expressed as Pearson coefficient; data are expressed as the mean ± SD of 3 independent experiments. Representative images of INSRβ intracellular localization in ADSC#5 treated with olanzapine and in control cells. INSRβ intracellular localization in RAB7-positive late endosomes after 16 h olanzapine treatment and 15 min insulin stimulation; INSRβ was stained using anti-INSRβ primary antibody and secondary Alexa Fluor 488 (green); RAB7 using anti-RAB7 primary antibody and secondary Alexa Fluor 546 (red) (**f**). Bar graph showing colocalization of INSRβ and RAB7 expressed as Pearson coefficient (**g**). Representative images of INSRβ localization in CD 63-positive exocytic vesicles after 16 h olanzapine treatment and 15 min insulin stimulation; INSRβ was stained using anti-INSRβ primary antibody and secondary Alexa Fluor 488 (green); CD63 using anti-CD63 primary antibody and secondary Alexa Fluor 546 (red) (**h**). Bar graph showing colocalization of INSRβ and CD63 expressed as Pearson coefficient (**i**). Representative images of INSR localization in lysosomes after 16 h olanzapine treatment and 15 min insulin stimulation. INSRβ was stained using anti-INSRβ primary antibody and secondary Alexa Fluor 488 (green); LAMP1 using anti-LAMP1 primary antibody and secondary Alexa Fluor 546 (red) (**j**). Bar graph showing colocalization of INSRβ and LAMP1 expressed as Pearson coefficient (**k**). Results are expressed as the mean ± SD of three independent experiments. White boxes indicates zoom area, white arrows indicates colocalization spots. *, Student’s *t*-test *p* < 0.05; **, Student’s *t*-test *p* < 0.01; ****, Student’s *t*-test *p* < 0.0001.

INSRβ endocytosis and turnover are crucial for modulating insulin signaling [43]. To investigate whether olanzapine disrupts this process, we conducted an endocytosis assay. ADSCs pretreated with 5 μM olanzapine for 16 h showed basal membrane INSRβ levels similar to the controls. However, following 15 min of insulin stimulation, we observed a significant reduction in INSRβ internalization (Figure 4a–e) and a significantly increased localization of INSRβ in RAB7-positive late endosomes (Figure 4f,g). At the same time, INSRβ localization in CD63-positive recycling endosomes was reduced, while colocalization in LAMP1-positive lysosomes was unaffected (Figure 4h–k).

Next, we assessed clathrin-dependent endocytosis (CDE) by measuring transferrin uptake, a well-established marker [44]. The results demonstrated a significant reduction in short-term transferrin endocytosis after 16 h of olanzapine treatment (Supplementary Figure S4), indicating that olanzapine may disrupt CDE pathways, which are crucial for the proper internalization of receptors, including INSR.

### 3.4. Olanzapine and Clozapine Induce Acidic Compartment Expansion

**Figure 5:**
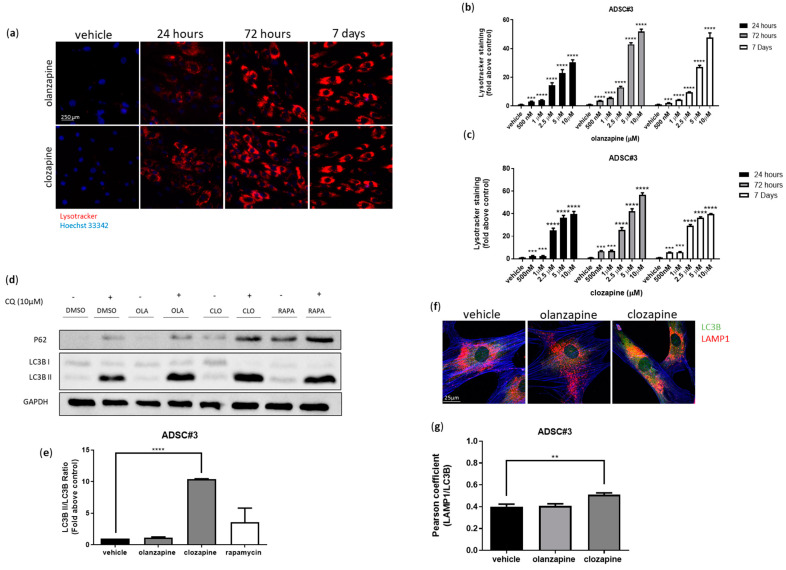

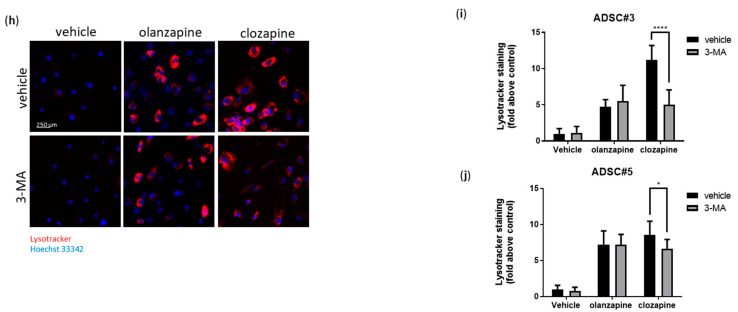
Olanzapine and clozapine induce expansion of intracellular acidic compartments and lysosomal biogenesis. Effects of olanzapine and clozapine on intracellular acidic compartments were evaluated by Lysotracker red staining and fluorescence microscopy after 24 h, 72 h, and 7 days of treatment. Nuclei were stained using Hoechst 33342. Representative images of ADSC#3 treated with vehicle (DMSO, negative control), 5 µM olanzapine, or clozapine at different time points (**a**). Graphs showing quantification of red Lysotracker staining/blue nuclei staining ratio as fold change relative to control; data are expressed as the mean ± SD of a representative experiment out of three independent experiments performed in triplicate (**b**,**c**). Representative image of WB analysis of ADSC#3 after 16 h treatment with SGAs; lysates were analyzed for LC3B, P62, and GAPDH (**d**). Bar graph showing quantification of the LC3B II/I ratio in ADSC#3 upon chloroquine treatment; densitometric analyses are expressed as the mean ± SD of three independent experiments performed in triplicate (**e**). Colocalization between LC3B (green) and LAMP1 (red) evaluated in ADSC#3 using confocal microscopy after 16 h treatment with vehicle, olanzapine, or clozapine (**f**). Histogram showing colocalization LAMP1/LC3B in ADSC#3 expressed as Pearson coefficient (**g**). Evaluation of intracellular acidic compartments, using Lysotracker red staining, in ADSC#3 cells after 16-h treatment with SGAs alone or in combination with 3-methyladenine (**h**). Bar graph showing acidic vesicle accumulation in ADSC#3 (**i**)) and ADSC#5 (**j**) treated for 16 h with olanzapine and 5 μM clozapine alone or in association with 3-methyladenine (3-MA); data are expressed as quantification of red Lysotracker staining/blue nuclei staining ratio as fold change relative to negative control. *, Student’s *t*-test *p* < 0.05; **, Student’s *t*-test *p* < 0.01; ***, Student’s *t*-test *p* < 0.001; ****, *p* < 0.0001.

Many psychotropic drugs from the cationic amphiphilic drug (CAD) family have been demonstrated to passively diffuse through lipid membranes and stack inside acidic vesicles such as lysosomes [45,46]. Given our previous findings that CADs disrupt lysosomal function in cancer cells [46], we investigated whether olanzapine and clozapine exhibit similar cationic amphiphilic properties in ADSCs. Lysotracker dye staining showed a progressive time- and dose-dependent increase in acidic compartment accumulation in ADSCs treated with olanzapine and clozapine (Figure 5a–c; Supplementary Figure S5). Notably, this effect was observed even in ADSCs treated with olanzapine and clozapine at concentrations below 1 µM, which are readily achievable in patients’ plasma and potentially in AT during chronic drug treatment [38,39]. Next, we investigated vacuolization and phospholipidosis induction, both of which are hallmark responses in cells exposed to CADs [47]. Although these phenotypes were prominently observed in ADSCs treated with the well-known CADs ebastine and fluoxetine, they were absent in those treated with olanzapine and clozapine (Supplementary Figure S6). Moreover, logP and PKa calculations [48] revealed that the values for olanzapine and clozapine fall outside the typical ranges observed for CADs (Table S1). This finding supports the hypothesis that olanzapine and clozapine influence acidic compartments through a mechanism distinct from that of CADs.

To further investigate the underlying mechanisms of acidic vesicle accumulation, we assessed autophagy induction in ADSCs following SGA treatment. The conversion of LC3B-I to LC3B-II was significantly increased after clozapine but not after olanzapine treatment (Figure 5d,e). Additionally, confocal microscopy revealed an increased colocalization of the lysosomal marker LAMP1 with LC3B in cells exposed to clozapine; this pattern was not observed in olanzapine-treated cells (Figure 5f,g). Lysotracker dye staining further demonstrated that acidic vesicle accumulation was significantly reduced in clozapine-treated ADSCs when combined with the PI3K class III inhibitor 3-MA, which impedes autophagy initiation. However, this reduction was not seen in cells treated with olanzapine (Figure 5h,i). These findings suggest that clozapine-induced vesicle accumulation is partially linked to autophagy, whereas olanzapine induces acidic compartment expansion through an autophagy-independent mechanism.

### 3.5. Olanzapine Promotes Lysosomal Biogenesis

**Figure 6:**
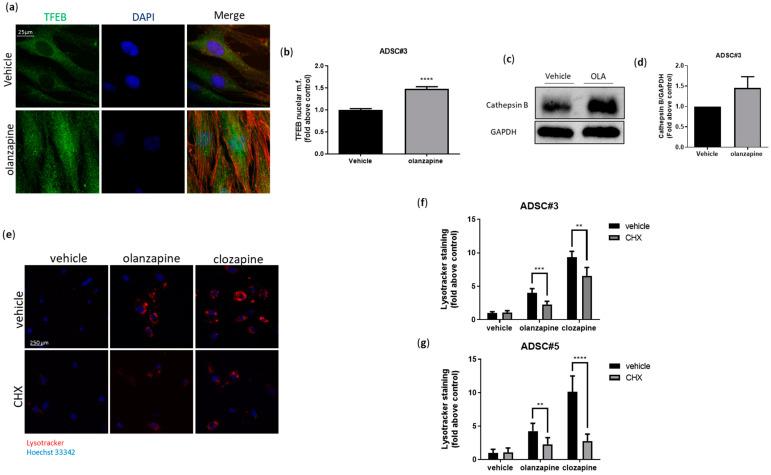
TFEB nuclear localization was investigated using confocal microscopy in ADSC#3 treated for 16 h with a vehicle or olanzapine. TFEB was stained using an anti-TFEB primary antibody and a secondary Alexa Fluor 488 (green), nuclei were stained using DAPI, and actin was stained using phalloidin 546 (**a**). Bar graph showing quantification of TFEB nuclear localization expressed as TFEB mean fluorescence in nuclear area normalized as fold change relative to control of three independent experiments (**b**). Representative images of WB analysis of ADSC#3 after 16-h treatment with olanzapine; lysates were analyzed for cathepsin B and GAPDH (**c**). Bar graph showing quantification of cathepsin B expression normalized on GAPDH. Densitometric analysis is expressed as the mean ± SD of three independent experiments (**d**). Evaluation of intracellular acidic compartments, using Lysotracker red staining, in ADSC#3 cells after 16-h treatment with SGAs alone or in combination with CHX (**e**). Bar graph showing acidic vesicle accumulation in cells treated for 16 h with olanzapine (5 µM) and clozapine (5 µM) alone or in the presence of CHX; data are expressed as quantification of red Lysotracker staining/blue nuclei staining ratio as fold change relative to negative control (**f**,**g**). **, Student’s *t*-test *p* < 0.01; ***, Student’s *t*-test *p* < 0.001, Student’s *t*-test ****, *p* < 0.0001.

To elucidate the mechanisms behind the autophagy-independent acidic compartment expansion induced by olanzapine and clozapine, we explored the role of transcriptional regulation, focusing on the Transcription Factor EB (TFEB). TFEB is a key regulator of lysosomal function and biogenesis. Its activity is enhanced by protein dephosphorylation, which leads to its translocation to the nucleus, where it drives the expression of genes essential for lysosomal synthesis, maturation, and degradation [49]. Our results demonstrated that ADSCs treated with 5 μM olanzapine for 16 h showed a 50% increase in TFEB nuclear localization and elevated levels of the TFEB-regulated lysosomal protease cathepsin B compared to untreated cells (Figure 6a–d). Additionally, when ADSCs were treated with SGAs in the presence of the protein synthesis inhibitor cycloheximide (CHX), there was a significant reduction in Lysotracker staining. These findings indicate that both olanzapine and clozapine stimulate lysosomal biogenesis through protein synthesis (Figure 6e,f).

### 3.6. PKCζ Dependency of SGA-Induced Lysosomal Expansion and Insulin Receptor Dysfunction

**Figure 7:**
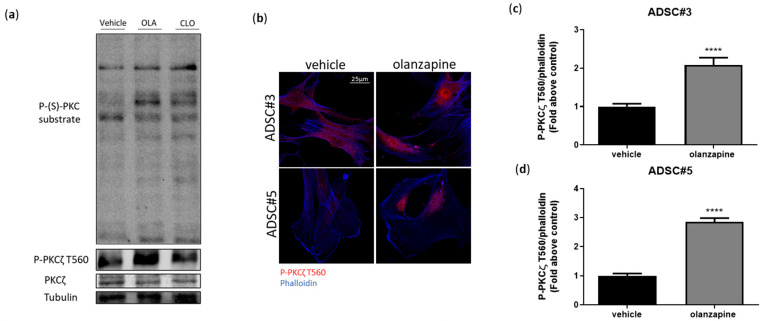

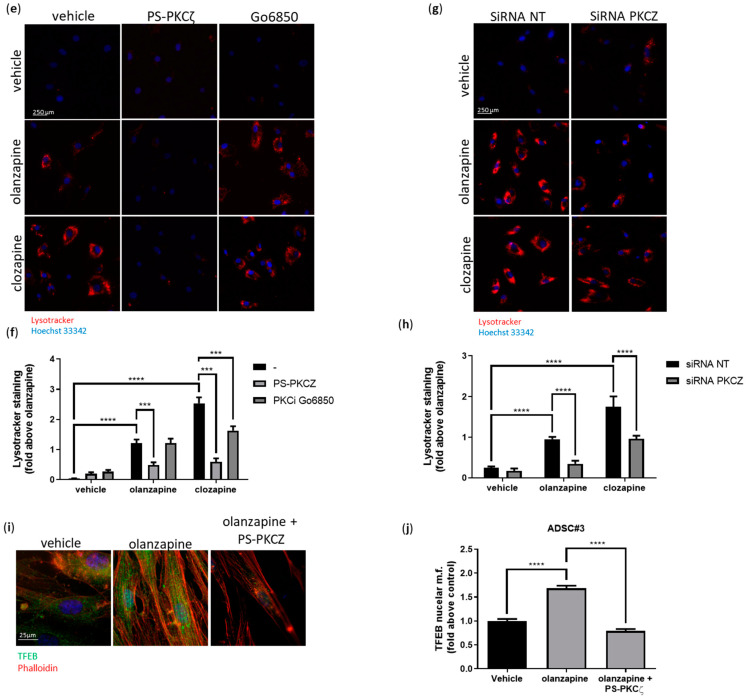
PKCζ-dependent expansion of acidic vesicles mediated by SGAs. Western blot analysis of ADSCs treated with 5 μM SGAs for 16 h. Lysates were analyzed for P-(Ser)-PKC substrate, P-PKCζ T560, total PKCζ, and tubulin (**a**). Confocal microscopy experiments showing P-PKCζ T560 localization in ADSC#3 treated with 5 μM olanzapine for 16 h (**b**). Bar graph showing quantification of P-PKCζ T560 normalized to cell area; data are expressed as the mean ± SD of three independent experiments (**c**,**d**). Evaluation of intracellular acidic compartments, based on Lysotracker red staining, in ADSC#3 cells after 16-h treatment with SGAs alone or in combination with Go6850 or PKCζ inhibitory pseudosubstrate (PS-PKCζ); nuclei were stained using Hoechst 33342 (**e**). Bar graph showing acidic vesicle quantification in cells treated for 16 h with olanzapine (5 μM) or clozapine (5 μM) alone, or in combination with Go6850 or PS-PKCζ; data are expressed as quantification of red Lysotracker staining/blue nuclei staining ratio as fold change relative to negative control and expressed as the mean ± SD of a representative experiment out of three independent experiments performed in triplicate (**f**). Representative images showing acidic vesicle accumulation in ADSC#3 transfected with SiRNA NT and SiRNA PKCζ and treated with olanzapine or clozapine for 16 h; nuclei were stained using Hoechst 33342 (**g**). Bar graph showing acidic vesicle quantification in ADSC#3 cells silenced for PKCζ and treated for 16 h with olanzapine or clozapine; data are expressed as quantification of red Lysotracker staining/blue nuclei staining ratio as fold change relative to negative control and expressed as the mean ± SD of a representative experiment out of three independent experiments performed in triplicate (**h**). Representative images of confocal microscopy analysis of TFEB localization using anti-TFEB primary antibody and Alexa Fluor 488 secondary antibody in ADSC#3 treated with olanzapine alone or in combination with PS-PKCζ (**i**). Bar graph showing quantification of TFEB nuclear localization expressed as TFEB mean fluorescence in nuclear area normalized as fold change relative to control of three independent experiments (**j**). ****, Student’s *t*-test *p* < 0.0001; ***, Student’s *t*-test *p* < 0.001.

Protein kinase C (PKC) enzymes, a family of serine/threonine kinases, are essential regulators of various cellular processes, including vesicular trafficking, lysosomal biogenesis, and tyrosine kinase receptor signaling [50,51]. Within this family, the atypical PKC (aPKCs) subfamily plays a pivotal role in insulin signaling and glucose uptake in the liver and AT [52,53,54]. Given the critical involvement of PKCs in processes disrupted by SGAs in ADSCs, we explored their role in the ADSCs’ response to SGA treatment. Western blot analysis revealed the activation of both canonical PKCs (cPKCs) and atypical PKCζ in ADSCs exposed to olanzapine and clozapine for 16 h (Figure 7a). Immunofluorescence further confirmed the activation and significant nuclear translocation of PKCζ, demonstrating a 2-fold increase in nuclear P-PKCζ T560 in olanzapine-treated ADSCs (Figure 7b–d).

To investigate whether PKC activation contributes to SGAs-induced acidic vesicle accumulation, we conducted Lysotracker experiments with olanzapine and clozapine, both alone and in combination with the canonical PKC inhibitor, Go6850, or the PKCζ-specific inhibitory pseudosubstrate (PS-PKCζ). The results showed that PS-PKCζ fully reverted acidic vesicle accumulation in both treatment conditions; in contrast, cPKC inhibition only partially reduced acidic vesicle accumulation in clozapine-treated cells (Figure 7e,f). Similar results were observed with PKCζ gene silencing (Figure 7g,h). Moreover, PS-PKCζ prevented the TFEB nuclear localization induced by olanzapine, indicating that PKCζ is required for TFEB-mediated acidic vesicle expansion (Figure 7i,j). Evidence showing that TFEB dephosphorylation can be modulated by protein phosphatase 2A (PP2A) [55,56], along with the association between PKCζ activation and PP2A activity, prompted us to examine lysosomal accumulation and TFEB translocation following PP2A inhibition with LB100. Our results showed a significant reduction in lysosomal accumulation and a complete abrogation of TFEB nuclear localization upon olanzapine treatment. This was similar to what was observed with PKCζ inhibition (Supplementary Figure S7), indicating that olanzapine-mediated TFEB activation requires PP2A activity.

**Figure 8:**
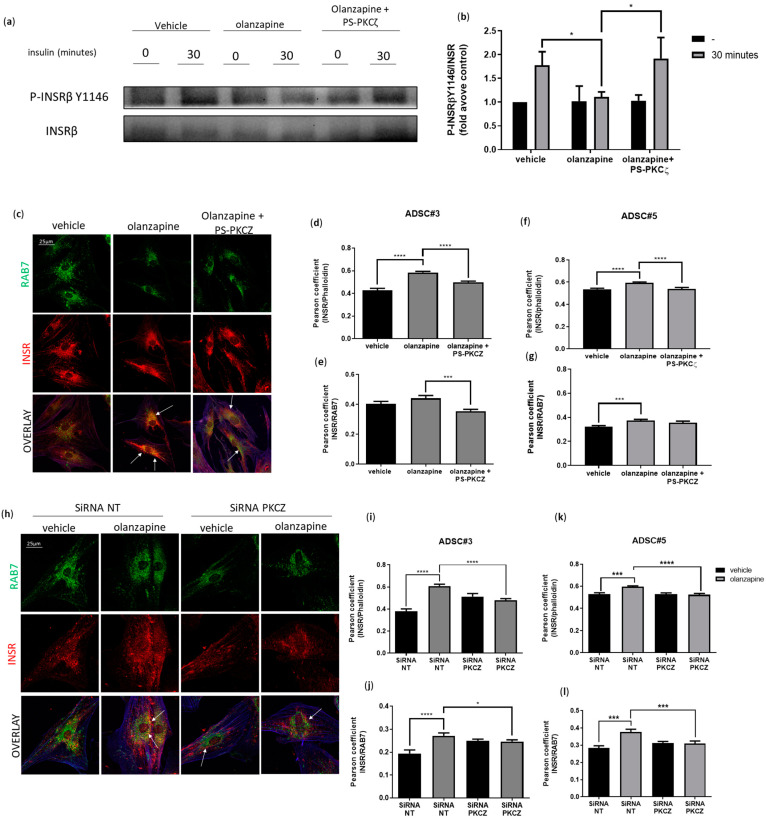
Olanzapine-induced metabolic alterations in ADSCs are PKCζ-dependent. Representative Western blot of ADSC#3 cells after 16 h of pretreatment with 5 µM olanzapine, either alone or in the presence of PS-PKCζ, followed by insulin stimulation (50 ng/mL) for 30 min. Lysates were analyzed for P-INSRβ Y1146 and total INSRβ (**a**). The bar graph shows the quantification of P-INSRβ Y1146 normalized to total INSRβ, expressed as fold change relative to control; data are presented as mean ± SD from three independent experiments (**b**). Representative images of ADSC#3 stimulated with insulin (50 ng/mL) following 16-h treatment with 5 μM olanzapine alone or in combination with PKCζ inhibitory pseudosubstrate showing INSRβ localization on the plasma membrane and in late endosomes; INSRβ was stained using anti-INSRβ primary antibody and secondary Alexa Fluor 546 (red); RAB7 was stained using anti-RAB7 primary antibody and secondary Alexa Fluor 488 (green); and actin was stained using phalloidin 633 (**c**). Bar graph showing colocalization of INSRβ and actin (**d**) or RAB7 (**e**) in ADSC#3 expressed as Pearson coefficient; data are expressed as the mean ± SD of three independent experiments. Bar graph showing colocalization of INSRβ and actin or RAB7 in ADSC#5 expressed as Pearson coefficient; data are expressed as the mean ± SD of three independent experiments (**f**,**g**). Representative images of ADSC#3 transfected with siRNA-targeting PKCζ and stimulated with insulin (50 ng/mL) after 16 h of treatment with 5 μM olanzapine. The images show INSRβ localization on the plasma membrane and within late endosomes; INSRβ was stained using anti-INSRβ primary antibody and secondary Alexa Fluor 546 (red); RAB7 was stained using anti-RAB7 primary antibody and secondary Alexa Fluor 488 (green); and actin was stained using phalloidin 633 (**h**). Bar graph showing colocalization of INSRβ and actin on plasma membrane expressed as Pearson coefficient in ADSC#3 and #5; data are expressed as the mean ± SD of three independent experiments (**i**,**k**). Bar graph showing quantification of colocalization of INSRβ with late endosome marker RAB7 expressed as Pearson coefficient in ADSC#3 and #5; results are expressed as the mean ± SD of three independent experiments (**j**,**l**). White arrows indicates colocalization spots. *, Student’s *t*-test *p* < 0.05; ***, Student’s *t*-test *p* < 0.001; ****, Student’s *t*-test *p* < 0.0001.

Next, we investigated the role of PKCζ in insulin signaling following olanzapine treatment. Western blot analysis showed that co-treatment with PS-PKCζ restored P-INSR Y1146 levels after 30 min of insulin stimulation, contrasting with olanzapine treatment alone (Figure 8a,b). AKT phosphorylation analysis consistently showed a similar rescue effect to that observed in INSR phosphorylation (Supplementary Figure S8). Confocal microscopy revealed that INSR retention at the plasma membrane during insulin stimulation was significantly diminished in olanzapine-treated ADSCs with either PKCζ silencing or exposure to PS-PKCζ (Figure 8c–l). Furthermore, INSR enrichment in RAB7-positive late endosomes was abolished in cells with PKCζ inhibition or gene silencing, underscoring PKCζ’s critical role in the abnormal internalization and turnover of INSR following olanzapine treatment (Figure 8c–l).

### 3.7. Olanzapine Activates PKCζ via GPCR Signaling

**Figure 9:**
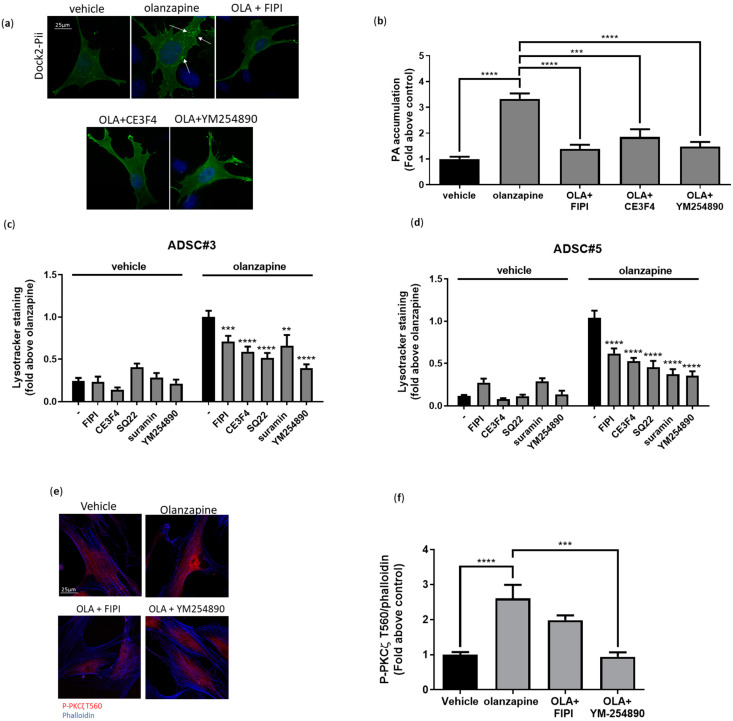
Olanzapine activates PKCζ by modulating GPCR signaling. PA accumulation was analyzed in 3T3L1 cells transfected with Pii-PA (PA indicator with superior sensitivity) DOCK2 (DOCK2-Pii) [34] after 16 h treatment with olanzapine alone or in combination with PLD inhibitor FIPI, EPAC inhibitor CE3F4, and Gq/11 inhibitor YM254890. Representative images of transfected cells treated with DMSO, negative control, 5 μM olanzapine alone or in combination with 750 nM FIPI. Arrows point to dots representing PA accumulation (**a**). Bar graph quantification of green dots normalized on cell area and expressed as fold change relative to control; results are expressed as the mean ± SD of three independent experiments (**b**). Bar graphs showing acidic vesicle accumulation in ADSC#3 (**c**) and ADSC#5 (**d**) treated for 16 h with olanzapine or clozapine (5 μM) alone or in combination with 750 nM Fipi, 10 μM EPAC inhibitor, 10 μM SQ22, 10 μM Suramin, or 10 μM YM254890; data are expressed as quantification of red Lysotracker staining/blue nuclei staining ratio as fold change relative to negative control and are expressed as the mean ± SD of a representative experiment out of three independent experiments performed in triplicate. Confocal microscopy assessment of P-PKCζ T560 expression in cells treated with 5 μM olanzapine/vehicle alone or in combination with 750 nM Fipi or 10 μM YM254890 for 16 h; phosphorylated PKC was evaluated using P-PKCζ T560 primary antibody and Alexa Fluor 546 secondary antibody, while actin was stained using Phalloidin 633 (**e**). Bar graph showing quantification of P-PKCζ T560 normalized to cell area; data are expressed as mean ± SD from three independent experiments (**f**). ** *p* < 0.01; *** *p* < 0.001; **** *p* < 0.0001 (Student’s *t*-test).

PKCζ is an atypical protein kinase C that becomes activated by directly binding to membrane phospholipids, such as phosphatidylinositol (3,4,5)-trisphosphate (PIP3) and phosphatidic acid (PA) [57]. To explore whether olanzapine-induced PKCζ activation is mediated through PA production, we measured PA levels in 3T3L1 cells transfected with a fluorescent plasmid encoding the PKC docking site responsible for PA binding (DOCK2-Pii). Our results revealed a significant accumulation of PA in olanzapine-treated cells compared to untreated controls (Figure 9a,b), suggesting that olanzapine may activate PKCζ by promoting PA production.

PA is primarily synthesized by phospholipase D (PLD) from phosphatidylcholine and by diacylglycerol kinases (DGKs) from diacylglycerol (DG) [58,59]. To determine whether PLD is upstream of olanzapine-induced PA accumulation, we treated ADSCs with olanzapine both alone and in the presence of the pan-PLD inhibitor, FIPI. Immunofluorescence showed significantly reduced PA (Figure 9a,b) and acidic vesicle accumulation (Figure 9c,d) in olanzapine-treated cells in the presence of FIPI, indicating the critical role of PLD in these processes. Conversely, treatment with DGK inhibitor R59022 did not affect PA levels or vesicle accumulation (Supplementary Figures S9 and S10), thereby ruling out DGKs as contributors to this mechanism.

PLD can be activated via downstream signaling pathways involving RTKs and GPCRs [60,61,62]. In a preliminary exploration of the signaling pathways upstream of the PKCζ/PLD axis, considering that olanzapine has an affinity for a large number of GPCRs and that there is no evidence suggesting interactions between SGAs and RTKs, we assessed intracellular PA levels following olanzapine treatment in combination with various inhibitors targeting GPCR-related pathways. Our results showed that PA levels were markedly reduced in cells treated with olanzapine in combination with either the EPAC inhibitor (CE3F4) or Gαq/11 inhibitor (YM254890) (Figure 9a,b). To characterize the pathway upstream of PLD, we performed LysoTracker staining on ADSCs treated with olanzapine, both alone and in combination with EPAC inhibitors (CE3F4), adenylate cyclase (SQ22), Gαq/11 (YM254890), and suramin. Our results demonstrated that all treatments significantly reduced the olanzapine-induced accumulation of acidic vesicles by at least 50%, with the strongest effect observed with YM254890. Additionally, fluorescence microscopy revealed that the inhibition of either PLD1 or Gαq/11 G proteins diminished PKCζ phosphorylation in olanzapine-treated cells (Figure 9e,f). These findings suggest that GPCR-related pathways, particularly those involving Gαq/11, play a crucial role in the olanzapine-mediated activation of the PKCζ/PLD axis and subsequent cellular responses.

## 4. Discussion

MetS is a pathological condition characterized by multiple metabolic abnormalities, including visceral obesity, hyperglycemia, hypertension, dyslipidemia, and IR [1,2]. The global rise in risk factors, especially obesity, longer lifespan, and unhealthy lifestyle choices, has driven the steady increase in MetS prevalence. The condition poses a significant public health threat due to its strong association with high-impact, chronic diseases like T2DM, CVD, and cancer [4,63]. Despite extensive investigation, the molecular mechanisms underlying MetS have been only partially clarified, and further research is necessary to optimize preventive and therapeutic strategies.

Recently, there has been growing interest in the role of AT disruption in the development of MetS [64,65,66], as well as in the involvement of ADSCs in AT pathophysiology [11,12,13,14]. These studies suggest that ADSC models are valuable for investigating the metabolic and molecular alterations in AT, particularly in vWAT, during MetS.

Various medications have notably been linked to an increased risk of MetS, IR, and T2DM, including glucocorticoids; protease inhibitors used in HIV treatment; beta-blockers; certain antidepressants, such as tricyclics and SNRIs; anticonvulsants like valproate and carbamazepine; and SGAs [67,68,69,70,71,72]. The pathogenic mechanisms underlying these risks are complex and may involve appetite dysregulation, energy expenditure, hepatic glucose metabolism, insulin secretion, and interference with adipocyte function and lipid metabolism [25,73]. SGAs have specifically been reported to significantly increase the risk of IR, T2DM, and MetS, even in the absence of weight gain, in patients undergoing chronic treatment [26,72,74], potentially through direct effects on AT [26,75,76]. Building on these premises, this study aimed to develop and characterize an in vitro vWAT model to investigate the molecular mechanisms underlying drug-induced MetS. The ultimate goal was to identify novel molecular pathways and potential druggable targets for MetS treatment. We utilized ADSCs isolated from human vWAT and exposed them to olanzapine and clozapine—two SGAs associated with a high risk of inducing IR, dyslipidemia, and T2DM within weeks of treatment [77].

Our findings were multifaceted. SGAs were shown to reduce insulin responsiveness, disrupt endolysosomal homeostasis, and impair adipogenic differentiation. Moreover, we observed that these effects were mediated by atypical PKCζ, potentially functioning downstream of GPCR signaling.

Firstly, we demonstrated that olanzapine treatment is associated with typical molecular hallmarks of IR [78,79,80]. Specifically, in cells treated with olanzapine, we observed a reduced phosphorylation of INSRβ Y1142, a marker of INSR activation, along with a decreased phosphorylation of key signaling proteins downstream of INSR upon insulin stimulation. Moreover, increased serine phosphorylation of INSR was observed, even in the absence of insulin stimulation. This finding links olanzapine treatment to alterations in insulin sensitivity, as the phosphorylation of INSR serine residues is a crucial event in the negative feedback regulation of insulin signaling; this is directly associated with the reduced phosphorylation of tyrosine residues and IR onset [56,81].

The inability of AT to properly respond to insulin leads to functional impairment, including reduced adipogenic differentiation [82]. Our findings demonstrated that when differentiating ADSCs were exposed to olanzapine at clinically relevant doses, lipid droplet formation, a critical indicator of adipogenesis, was reduced. This finding contrasts with other studies reporting enhanced adipogenesis with SGA exposure [83,84,85]. Such discrepancies may stem from differences in the models used—often murine or human subcutaneous ADSCs—which differ from vADSCs in their function, adipogenic potential, and pharmacological response [7,31,32,86]. Furthermore, many prior studies used substantially higher drug concentrations, potentially activating alternative pathways that contribute to lipid accumulation due to the complex pharmacological profiles of psychotropic drugs.

Our findings revealed that SGA treatment leads to a loss of lysosomal compartment homeostasis, resulting in the accumulation of acidic vesicles in a time- and dose-dependent manner. This starts from drug concentrations that are readily achieved in patients undergoing chronic treatment [38,39]. This phenomenon is commonly observed with many psychotropic drugs, which are known for their cationic amphiphilic properties that enable them to accumulate in acidic organelles such as lysosomes. Such accumulation disrupts the normal physiological functions of these cellular components and may lead to cell death through mechanisms like lysosomal membrane permeabilization [87,88]. However, neither olanzapine nor clozapine caused vacuolization or phospholipidosis, which are typical phenotypes associated with CADs [47]. Furthermore, based on Muehlbacher et al.’s classification system [48], which uses LogP and pKa values to categorize CADs, neither olanzapine nor clozapine meets the criteria for CADs. These observations suggest that both drugs likely exert their effects on ADSCs through mechanisms distinct from those of compounds with a general affinity for acidic vesicles.

Psychotropic drugs have been noted for their ability to modulate lysosomal function across various models [89,90], with experimental evidence suggesting that these drugs may disrupt endolysosomal compartments and potentially cause a late-stage block in autophagic flux [91]. In our study, a Western blot analysis of LC3BI to LC3BII conversion and confocal microscopy of LC3B/LAMP1 colocalization did not reveal autophagy induction following olanzapine treatment; the same was not true for clozapine. The ineffectiveness of the PI3K class III inhibitor 3-MA in reducing vesicle accumulation induced by olanzapine, combined with its partial effect on clozapine-treated cells, confirmed that the drug’s impact on lysosomal compartments is independent of autophagy. In contrast, clozapine’s effect on acidic compartments involves both autophagy-dependent and independent mechanisms. The autophagy-dependent acidic vesicle expansion induced by clozapine aligns with previous reports [92] and possibly reflects a protective response to clozapine-induced mitochondrial or endoplasmic reticulum (ER) stress to mitigate energy depletion and/or ER dysfunction [93,94]. This process may occur independently of, and concurrently with, the autophagy-independent mechanisms induced by olanzapine.

In our model, we demonstrated that lysosome formation drives the autophagy-independent expansion of the acidic compartment induced by SGAs. These findings unveil a novel mechanism whereby SGAs promote lysosomal biogenesis through TFEB activation mediated by the PP2A/PKCζ pathway.

Under physiological conditions, the phosphorylation of serine residues on TFEB promotes binding to TFE3, resulting in retention in the cytosol [56]. TFEB can be activated by protein phosphatase 2A (PP2A), which is then dephosphorylated, enabling its nuclear translocation and the subsequent initiation of the transcriptional program for lysosomal biogenesis [9,56]. Our study demonstrated that the inhibition of aPKC or PP2A effectively prevents TFEB nuclear translocation and lysosomal biogenesis. Olanzapine-induced lysosomal expansion has previously been reported in insulin-sensitive tissues, such as liver and subcutaneous AT from obese mice, where it was associated with enhanced autophagy and metabolic effects that can be rescued to restore the physiological autophagic flux [95]. Conversely, in human liver cell lines, this phenomenon has been interpreted as an autophagic blockade due to cholesterol accumulation in lysosomes [96], though the underlying mechanisms were not explored further. Notably, a relationship between SGAs, TFEB activation, and lysosomal biogenesis has been observed in C. elegans neurons exposed to olanzapine, in which the treatment results in impaired lysosomal/autophagosomal fusion and lysosome accumulation [97], and in human microglial cells treated with clozapine, which induced TFEB nuclear translocation [98]. However, the specific mechanisms remain to be elucidated in these models.

PKCs are a family of proteins that play a direct role in endolysosomal homeostasis, lysosomal biogenesis, and endocytic traffic regulation [99,100,101,102]. PKCs, including atypical PKCζ, have been shown to interact with and regulate PP2A in studies involving NIH-3T3 cells under SV40 small t stimulation [81] and rat brain microvascular endothelial cells treated with endothelial monocyte-activating polypeptide-II (EMAP-II) [103]. However, to the best of our knowledge, no data are currently available regarding their role in AT. aPKCs also participate in the downregulation of INSR signaling by impairing AKT activity and insulin-induced gene expression [104,105,106]. In particular, PKCζ is involved in the regulation of glucose uptake and vesicular trafficking in insulin-sensitive tissues [107,108,109]. Additionally, PKCζ has been shown to interact with INSR and actively downregulate physiological insulin signaling by phosphorylating IRS1 serine residues [110,111,112,113,114,115]. Our data demonstrate that, in olanzapine-treated cells, PKCζ inhibition or silencing not only reversed acidic compartment expansion but, more importantly, fully restored INSR tyrosine phosphorylation and turnover in response to insulin stimulation. Our findings align with the existing literature and reveal, for the first time, the central role of PKCζ in mediating olanzapine-induced alterations in cellular and metabolic processes in ADSCs, contributing to the onset of IR.

We also found that olanzapine treatment affects clathrin-mediated endocytosis (CME), as evidenced by reduced transferrin internalization. These findings align with data from Daniel et al. [116] demonstrating that antipsychotics from the phenothiazine family, such as chlorpromazine, selectively inhibit dynamin 1 GTPase and disrupt CME. However, SGAs like clozapine, olanzapine, and risperidone have a less well-defined effect on dynamin 1, with the underlying mechanism still not fully understood [116]. On the other hand, compromised CME has been observed in type A Niemann–Pick Disease and other lysosomal storage disorders, where intracellular lysosomal accumulation leads to aberrant vesicular trafficking and impaired endocytosis [117,118]. These observations suggest that, in our ADSC model, while a direct effect of SGAs on the CME pathway cannot be ruled out, defective INSR internalization is likely a consequence of both PKCζ-dependent lysosomal compartment expansion and impaired INSR tyrosine phosphorylation, which is essential for INSR internalization and turnover [43,117,118,119,120].

Recognizing the key role of PKCζ in mediating the effects of olanzapine in ADSCs, we conducted a preliminary investigation into the signaling pathways upstream of this kinase. While canonical and novel PKCs are activated by calcium and/or DG, atypical PKCs, including PKCζ, are activated by phospholipids, particularly by PA [121,122]. We confirmed that olanzapine induces PA production and determined that PLDs are largely responsible for this production downstream of olanzapine.

PLDs can be activated by various signaling pathways, either downstream of GPCRs via small GTPases such as EPAC, ARF, and RhoA [123,124], or through RTKs [60,61,62]. Given olanzapine’s high affinity for GPCR family receptors [125], we investigated the PLD signaling pathway downstream of these receptors. Our results unveiled that the EPAC inhibitor significantly reduced, but did not completely abolish, the olanzapine-induced accumulation of acidic vesicles. EPAC is an effector of cAMP produced by adenylate cyclase (AC), which is typically activated by GPCRs coupled to Gas [126]. Our data showed that treatment with the AC inhibitor or suramin significantly, though not entirely, reduced the Lysotracker signal. These observations suggest the primary, but not exclusive, role of the cAMP/EPAC signaling pathway in PKCζ-dependent acidic vesicle expansion. Notably, another major source of PA are DGKs; these are lipid kinases that play a central role in many lipid signaling pathways by converting DG to PA [127,128]. However, DGK involvement appears unlikely, as the DGK inhibitor, R59022, was ineffective in reducing the lysosomal biogenesis induced by olanzapine.

Notably, a significant reduction in olanzapine-induced acidic vesicle accumulation was observed with YM254890. While a recent study showed that YM254890, in addition to its potent Gq/11 inhibitory function, can act as a suramin analog by blocking Gas receptors at concentrations below 10 μM [129], this observation does not exclude the potential role of Gαq/11 signaling in the PKCζ-dependent acidic vesicle accumulation and insulin resistance induced by SGAs. The activation of calmodulin pathway downstream calcium signaling and PKCα activation was demonstrated to, in turn, activate PLDs [130]. Evidence indicates that calcium signaling can activate calcium-dependent AC via CaM/CaMK [131,132], whereas AC5 has been shown to be activated downstream of Gaq [133,134]. Collectively, these results support the hypothesis that olanzapine activates atypical PKC through GPCR signaling involving Gaq and the cAMP/EPAC/PLD axis. However, pharmacological EPAC and PLD inhibition does not fully reverse the accumulation of acidic vesicles, whereas PKCζ inhibition almost completely rescues this phenotype. This suggests that additional mechanisms may converge to activate PKCζ. We cannot rule out the possibility that other phospholipids, such as ceramides, may be involved in olanzapine-mediated PKC activation [135]. Further investigations are needed to fully elucidate this mechanism.

**Figure 10:**
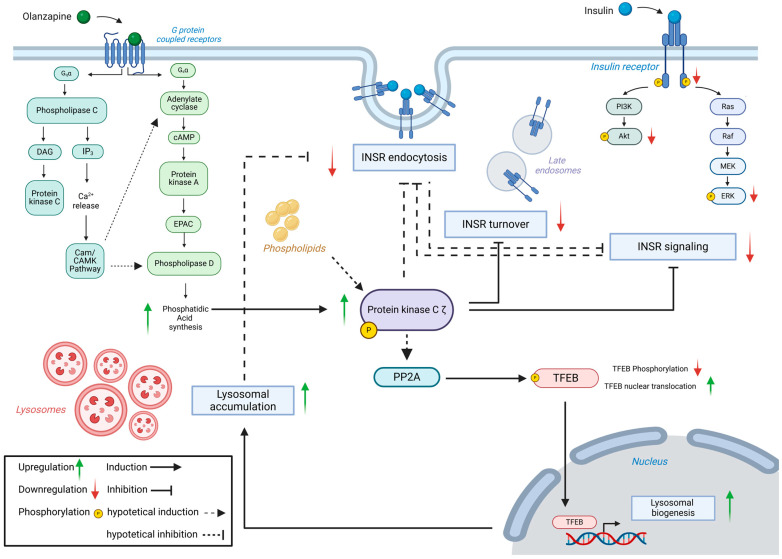
Proposed mechanism of olanzapine-induced metabolic disruption. Olanzapine’s effects are mediated by Gαq and Gαs, initiating signaling cascades that activate phospholipase D and PKCζ. PKCζ disrupts insulin signaling and impairs INSR turnover. Through PP2A activation, this leads to TFEB dephosphorylation and nuclear translocation, promoting lysosomal biogenesis. The combined effects of lysosomal accumulation and PKCζ-induced disruption of INSR phosphorylation further impair insulin signaling and INSR turnover. Image created in https://BioRender.com (accessed on 30 October 2024).

In conclusion, our findings demonstrate that SGAs disrupt lysosomal biogenesis and insulin sensitivity in ADSCs derived from human vWAT depots through a mechanism involving PKCζ signaling downstream of GPCR activation (Figure 10). Our results are consistent with existing evidence that SGAs can impact lipid and glucose metabolism in various in vitro and in vivo models [77,136,137] and confirm the translational value of ADSCs as a reliable in vitro model for studying drug-induced vWAT alterations. This study also identified aPKC as a potential therapeutic target for drug-induced MetS. Atypical PKC inhibitors have been tested not only in preclinical in vivo models but also in clinical trials for medical conditions like chronic pain, mental health disorders, and cancer [138,139,140,141,142]. Although these compounds have not been tested for metabolic disorders, the insights presented in our work could serve as a foundation for future research exploring their application for these conditions [143]. SGAs significantly affect other tissues such as the muscles and liver [144]. Extending our findings to these tissues will further elucidate the molecular pathways underlying the systemic effects of SGAs, offering deeper insights into their broader metabolic impact. Ultimately, investigating the molecular mechanisms driving drug-induced metabolic dysregulation offers valuable opportunities to uncover alterations linked to MetS and identify novel druggable targets, paving the way for more effective therapeutic strategies for these patients.

## Figures and Tables

**Table 1:** Oligo sequences (5′–3′) for the investigated genes.

Target Gene	Forward	Reverse
PPARG	TCAGGTTTGGGCGGATGC	TCAGCGGGAAGGACTTTATGTATG
FABP4	TCAGTGTGAATGGGGATGTGAT	TCTGCACATGTACCAGGACACC
LPL	CATTCCCGGAGTAGCAGAGT	GGCCACAAGTTTTGGCACC
HSL	GACCACTCCAACTCAGACCA	GGGTCAGGTTCTTGAGGGAA

## Data Availability

The raw data supporting the conclusions of this article will be made available by the authors upon request.

## Supplementary Materials

The following supporting information can be downloaded at https://www.mdpi.com/article/10.3390/cells13242084/s1. Figure S1: ADSCs express stemness markers. Figure S2: Cytotoxic activity of SGAs. Figure S3: Olanzapine affects ADSCs adipogenic differentiation. Figure S4: Olanzapine inhibits clathrin-dependent endocytosis. Figure S5: SGAs induces acidic vesicle formation. Figure S6: Olanzapine and clozapine do not display cationic amphiphilic characteristics. Figure S7: PP2A inhibition rescues TFEB-mediated lysosomal synthesis. Figure S8: Olanzapine-induced metabolic alterations in ADSCs are PKCζ-dependent. Figure S9: Olanzapine-induced PA accumulation is not DGK-dependent. Figure S10: Olanzapine-mediated acidic vesicle accumulation is not DGK-dependent. Table S1: Evaluation of cationic amphiphilic properties of antipsychotics.
