# Supplementary material for: Second-Generation Antipsychotics Induce Metabolic Disruption in Adipose Tissue-Derived Mesenchymal Stem Cells Through an aPKC-Dependent Pathway
Source: Cells. 2024 Dec 17;13(24):2084. doi: 10.3390/cells13242084 (PMC11674800; doi:10.3390/cells13242084)
Supplement: Supplementary file 1 [file cells-13-02084-s001.zip › cells-3269248-Supplementary materials.pdf]

Supplementary Figures

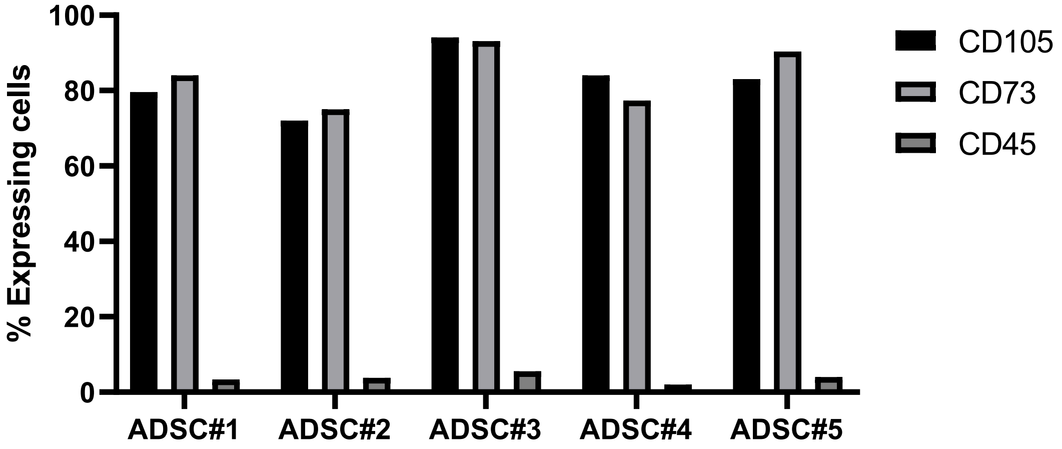

Supplementary Figure S1. ADSCs express stemness markers. Representative histogram showing mesenchymal stem cells markers CD105, CD73 and haemopoietic marker CD45 in ADSCs.

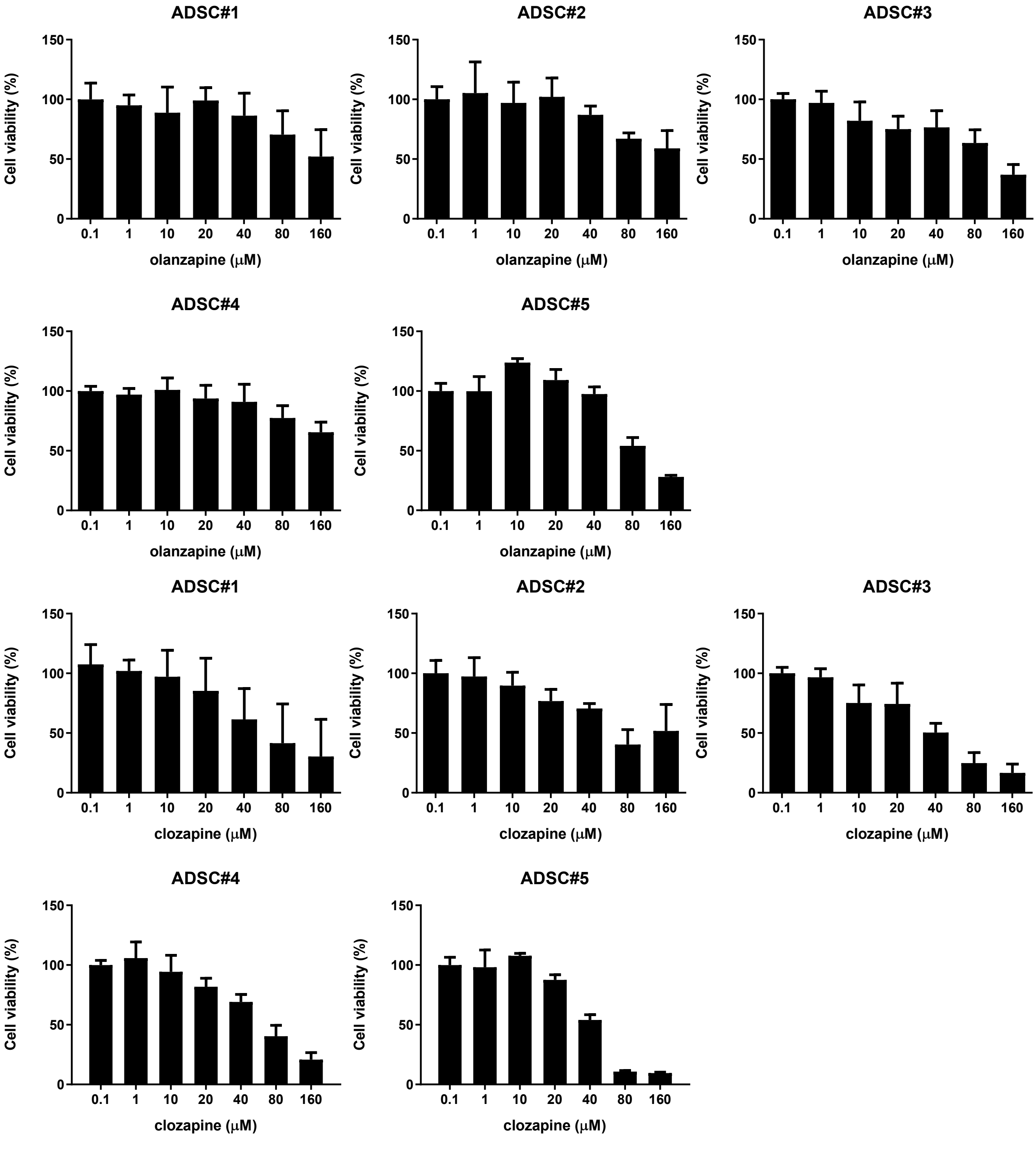

Supplementary Figure S2. Cytotoxic activity of SGAs. Five different ADSCs lines were treated for 72h with scalar doses of drugs. Viabilities were assessed by Cell Tox Green assay. Data are presented as mean ± standard error of the mean (SEM) from three-five independent experiments.

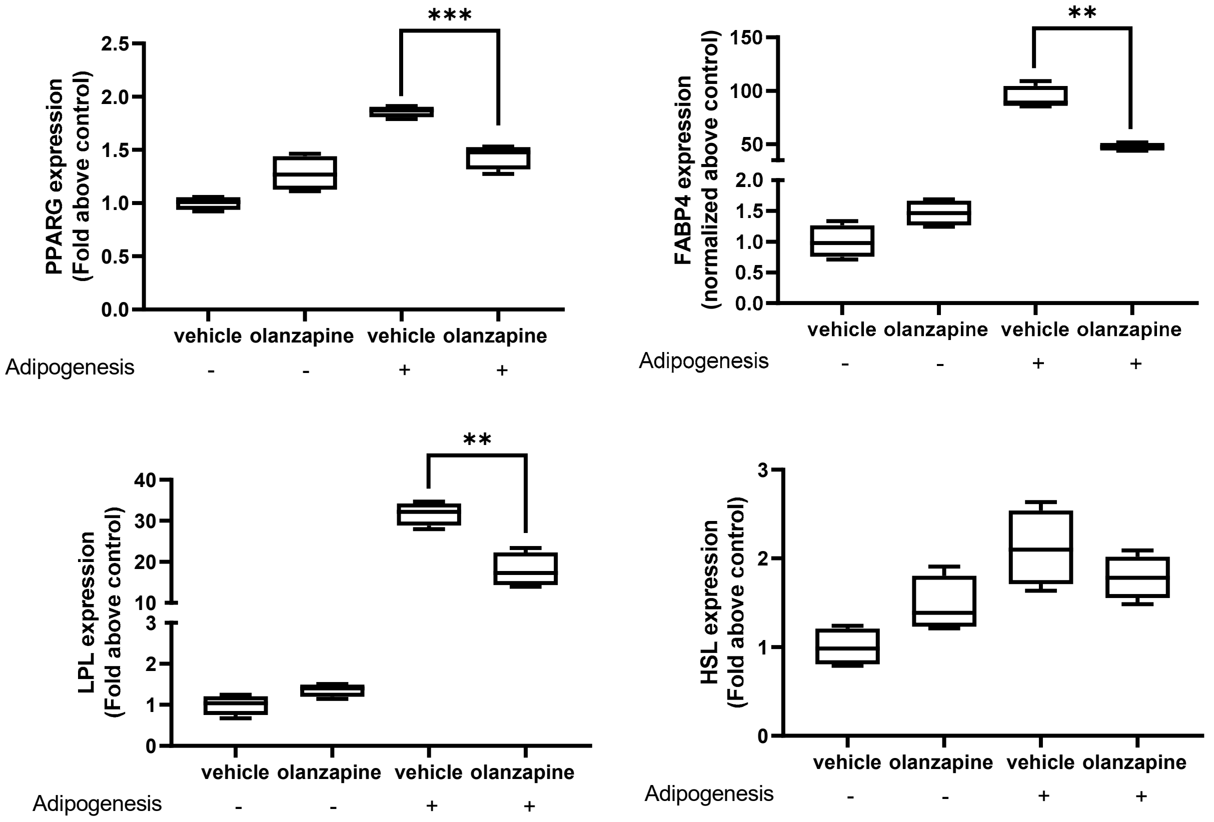

Supplementary Figure S3. **Olanzapine affects ADSCs adipogenic differentiation** Graphs showing expression of adipogenic genes PPARG (a), FABP4 (b), LPL (c) and HSL (d). Graphs are expressed as the mean ± standard deviation of three independent experiments and normalized as fold change relative to control. **, Student's T-test $p < 0.01$ ***, Student's T-test $p < 0.001$.

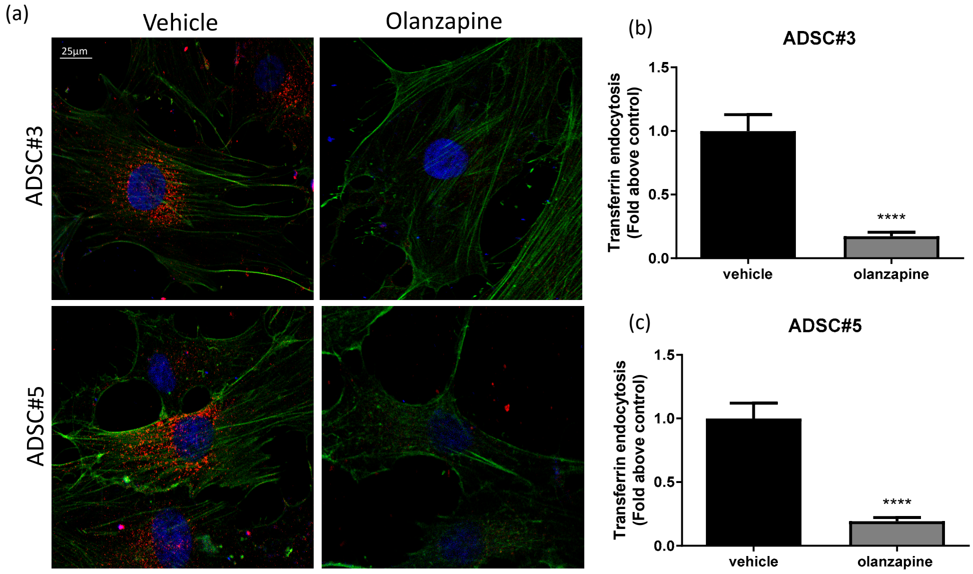

Supplementary Figure S4. Olanzapine inhibits clathrin-dependent endocytosis. Representative images of ADSC#3 and ADSC#5 treated for 16h with olanzapine and stimulated for 5min with transferrin Texas red (25µg/ml); actin was stained using phalloidin 488, nuclei were stained using DAPI (**a**). Histograms showing quantification of internalized transferrin after 5min stimulation; internalization was quantified normalizing red fluorescent signal on cell area as fold change relative to control and all results are expressed as the mean ± SD of three independent experiments (**b, c**). **** Student's T-test $p < 0.0001$.

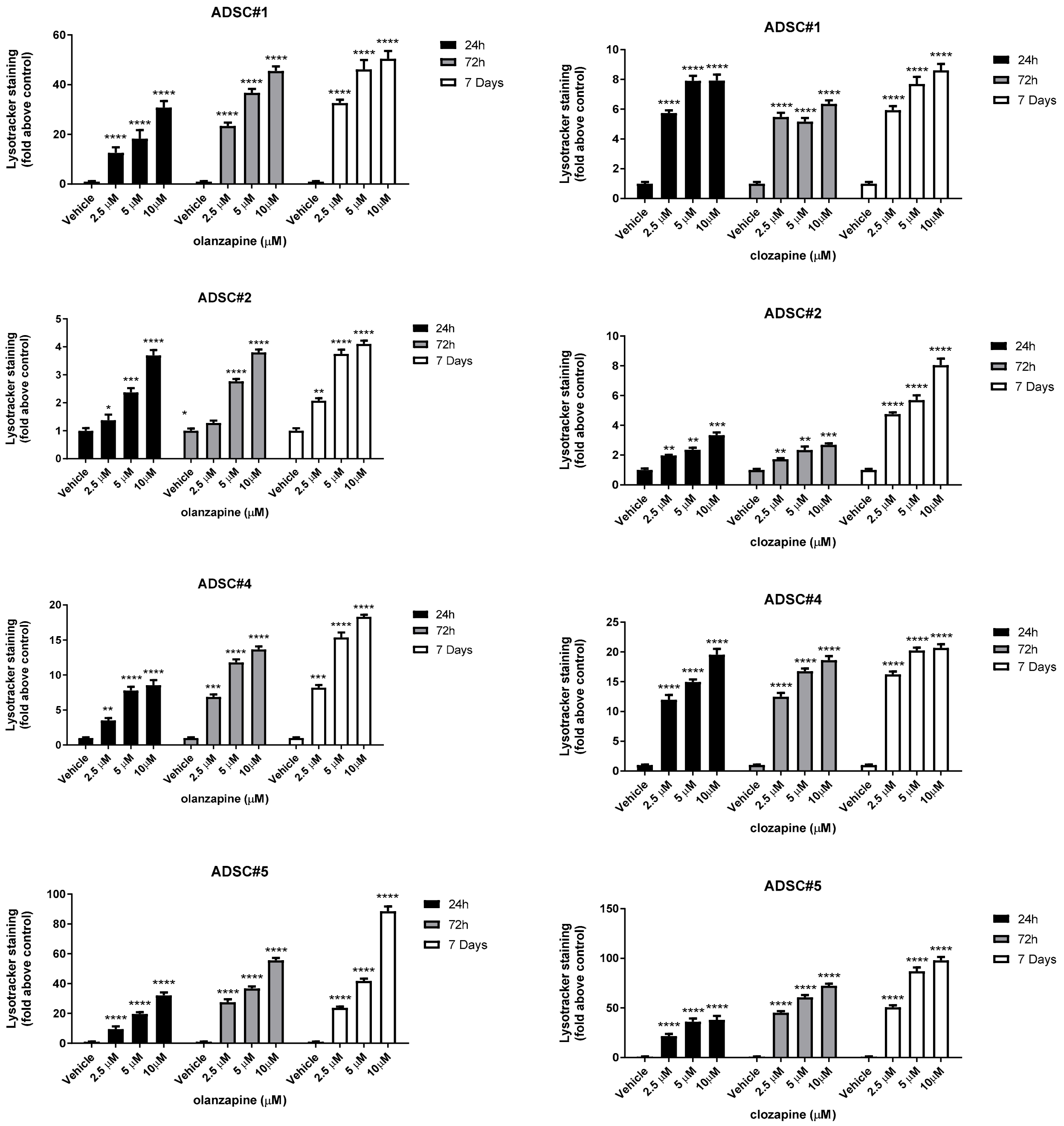

Supplementary Figure S5. SGAs induces acidic vesicles formations. Effects of olanzapine and clozapine on intracellular acidic compartments were evaluated by Lysotracker Red staining after 24, 72h and 7 days of treatment. Nuclei were stained using Hoechst 33342. Pictures were acquired by fluorescence microscopy. Graphs showing quantification of red Lysotracker staining/blue nuclei staining ratio as fold change relative to negative control. Data are expressed as the mean ± SD of a representative experiment out of three independent experiments performed in triplicate. ****, Student's T-test $p < 0.0001$.

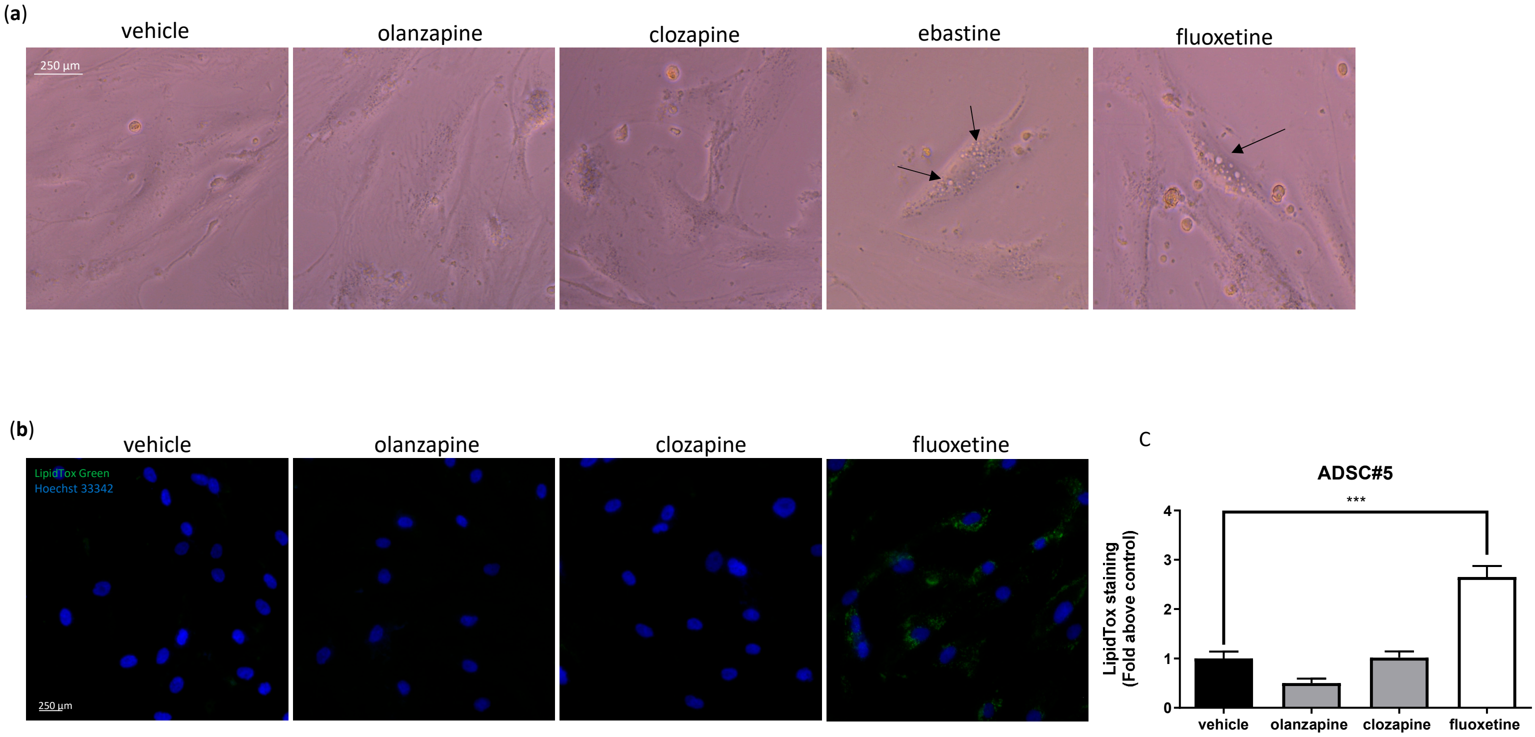

Supplementary Figure S6. Olanzapine and clozapine do not display cationic anphiphilic characteristics. Representative images showing the assessment of cytoplasmic vacuolization by phase contrast microscopy in ADSC#3 treated with vehicle (DMSO, negative control), olanzapine, clozapine, or positive controls ebastine and fluoxetine; arrows point the vesicles formation (**a**). Representative images showing the evaluation of phospholipids in ADSC#5 after 16h treatment with 5μM olanzapine, clozapine, fluoxetine (positive control), or vehicle, by LipidTox green staining and fluorescence microscopy; nuclei were stained using Hoechst 33342. (**b**). Histogram showing quantification of Green LipidTox staining/blue nuclei staining ratio as fold change relative to control; data are presented as mean ± SD from three independent experiments, each performed in triplicate (**c**). ***, Student's T-test $p < 0.001$

Supplementary 2 TABELLA CADs

Compound	MetS risk	LogP (ACD Labs)	pKa (ACD Labs)	CADs classification*
aripiprazole	low	5.6	6.52	NO
Ziprasidone	low	4	4.01	NO
Paliperidone	Low	1.52	7,89	NO
quetiapine	intermediate	1.57	7.06	NO
risperidone	intermediate	2.89	7.91	NO
chloropromazine	intermediate	5.2	9.41	YES
**olazapine**	**high**	**1.78**	**10.57**	**NO**
**clozapine**	**high**	**2.36**	**7.5**	**NO**
fluoxetine	-	4.09	10.06	YES
ebastine	-	7.79	8.48	YES
pimozide	-	6.38	9.42	YES
fluspirilene	-	3.89	9.21	YES
penfluridol	-	6.01	8.4	YES
amiodarone	-	8.89	9.37	YES
chloroquine	-	4.69	10.47	YES

Supplementary Table S1. Evaluation of cationic amphiphilic properties of antipsycotics by calculation of LogP and pKa (LogP > 3, for the amphiphilic characteristics, and a PKa > 7.4 for the cationic characteristics).

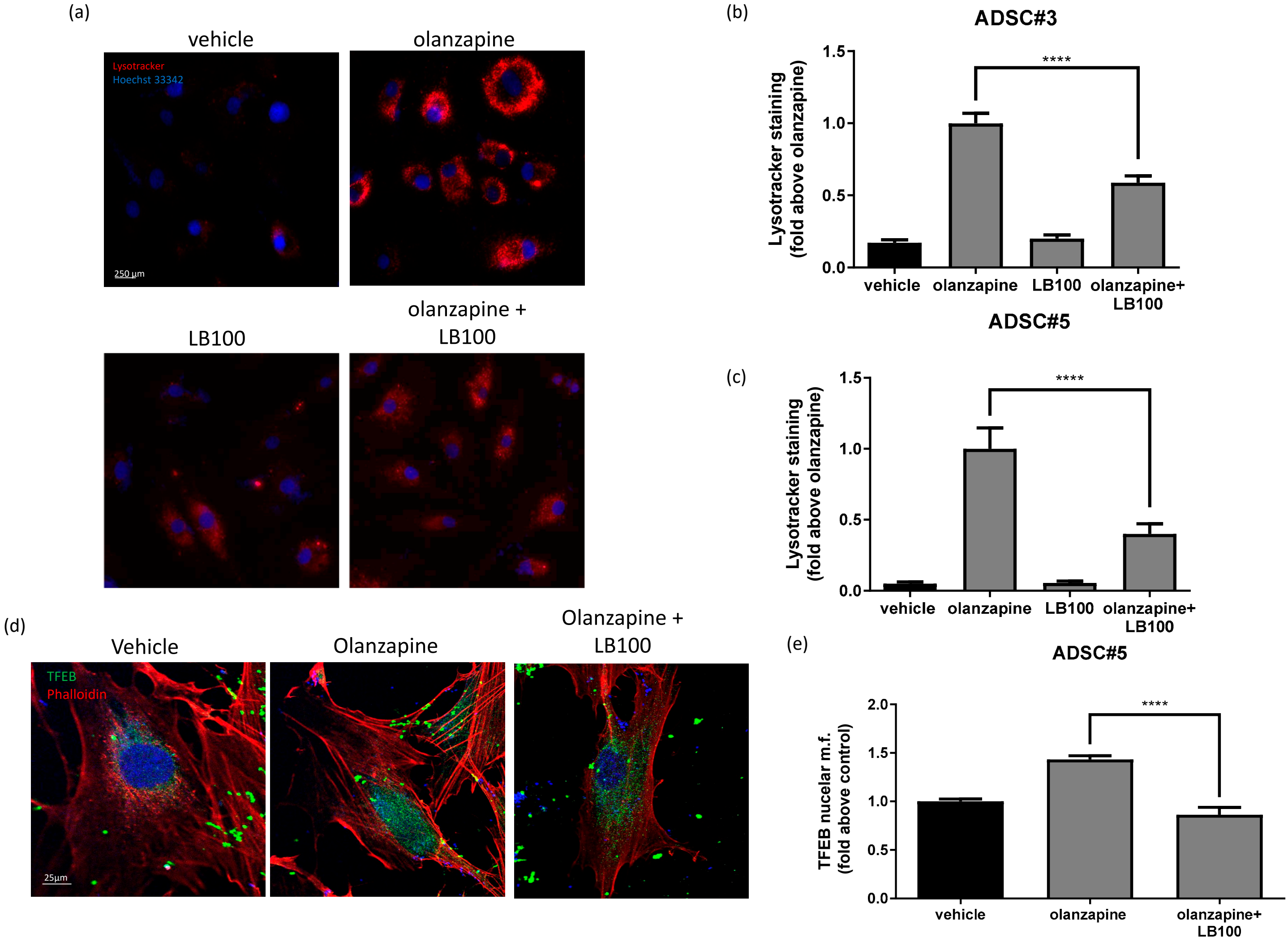

Supplementary Figure S7. PP2A inhibition rescue TFEB mediated-lysosomal synthesis. Evaluation of intracellular acidic compartments, by Lysotracker Red staining, in ADSC#3 and ADSC#5 cells after 16 hours treatment with SGAs alone or in combination with LB100 (1 μM); nuclei were stained using Hoechst 33342 (**a**). Histogram showing acidic vesicles quantification in cells treated for 16h with olanzapine (5μM) alone, or in combination with Go6850 or PS-PKCζ; data are expressed as quantification of red Lysotracker staining/blue nuclei staining ratio as fold change relative to negative control and expressed as the mean ± SD of a representative experiment out of three independent experiments performed in triplicate (**b, c**). Representative images of confocal microscopy analysis of TFEB localization by using anti-TFEB primary antibody and Alexa Fluor 488 secondary antibody in ADSC#3 treated with olanzapine alone or in combination with LB100 (**d**). Histogram showing quantification of TFEB nuclear localization expressed as TFEB mean fluorescence in nuclear area normalized as fold change relative to control of three independent experiments (**e**). **** Student's T-test $p < 0.0001$.

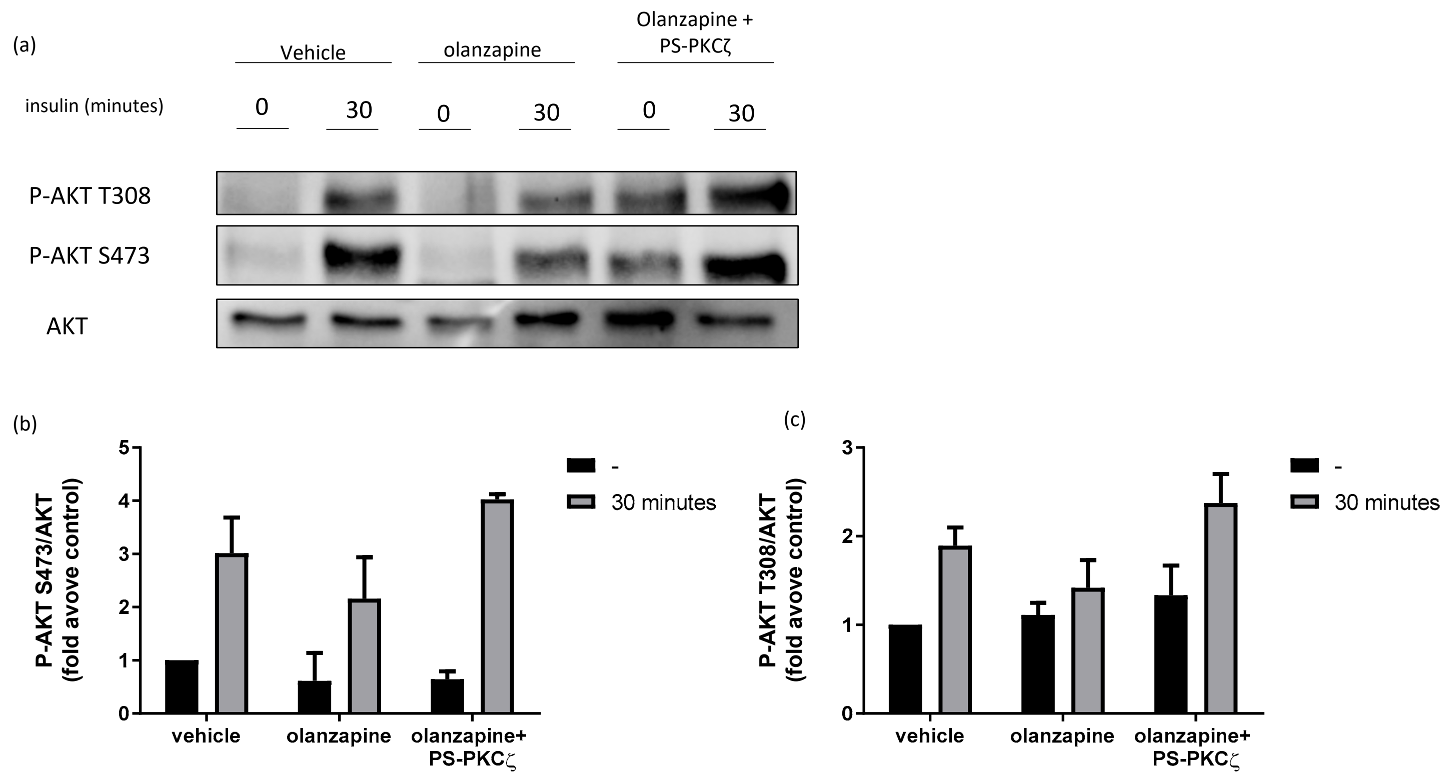

Supplementary Figure S8 . Olanzapine-induced metabolic alterations in ADSCs are PKCζ dependent. Representative Western blot of ADSC#3 cells after 16 hours pretreatment with 5μ olanzapine alone or in the presence of PS-PKCζ and stimulation with insulin (50ng/ml) for 30 minutes. Lysates were analyzed for P-AKT T 308, P-AKT 473 and total AKT (a). Histogram showing quantification of P-INSRβ Y1142 normalized on total INSRβ and expressed as fold change relative to control, Graphs are expressed as the mean ± SD of three independent ex-periments. (b,c).

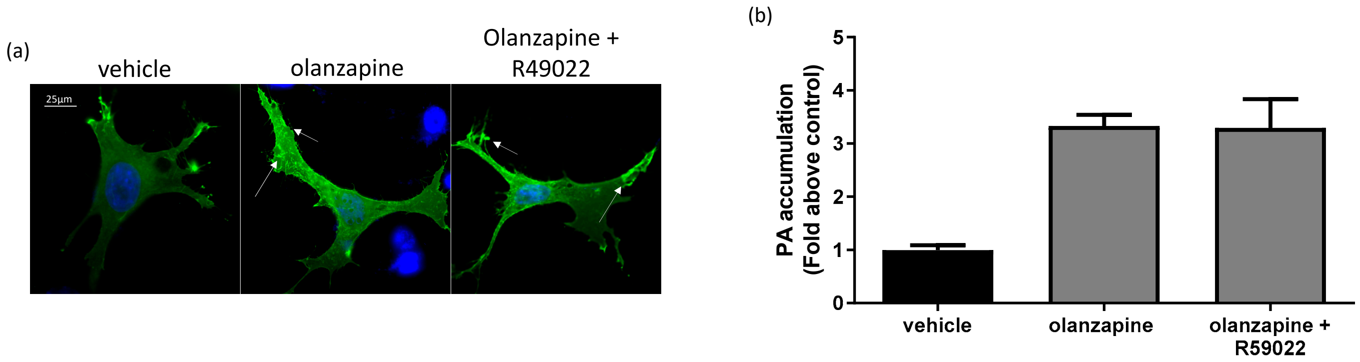

Supplementary Figure S9. **Olanzapine induced PA accumulation is not DGK dependent**. PA accumulation was analyzed in 3T3L1 cells transfected with Pii-PA (PA indicator with superion sensitivity) DOCK2 (DOCK2-Pii) (Zhang 2013). Representative images of transfected cells treated with DMSO, negative control, 5µM olanzapine alone or in combination with DGK inhibitor R59022 (**a**). Histogram showing quantification of green dots normalized on cell area and expressed as fold change relative to control (**b**); results are expressed as the mean ± SD of three independent experiments

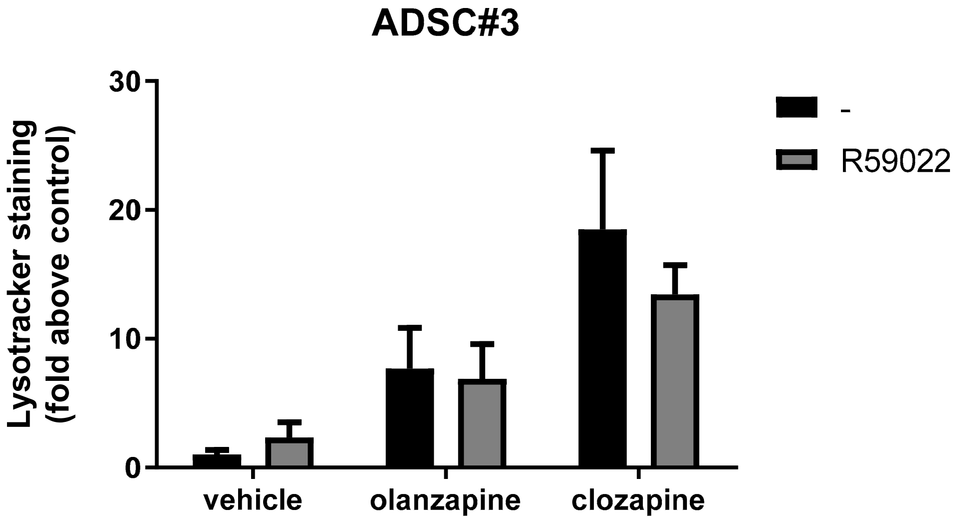

Supplementary Figure S10. **Olanzapine-mediated acidic vesicles accumulation is not DGK dependent.** Histogram showing acidic vesicles accumulation in ADSC#3 treated for 16 hours with olanzapine and clozapine (5µM) alone or along with R59022 10µM. Data are expressed as quantification of red lysotracker staining/blue nuclei staining ratio as fold change relative to negative control, Data are expressed as the mean ± SD of a representative experiment out of three independent experiments performed in triplicate
